# Determinants of Serum Immunoglobulin Levels: A Systematic Review and Meta-Analysis

**DOI:** 10.3389/fimmu.2021.664526

**Published:** 2021-04-07

**Authors:** Samer R. Khan, Anna C. van der Burgh, Robin P. Peeters, P. Martin van Hagen, Virgil A. S. H. Dalm, Layal Chaker

**Affiliations:** ^1^ Department of Epidemiology, Erasmus University Medical Center, Rotterdam, Netherlands; ^2^ Department of Internal Medicine, Division of Clinical Immunology, Erasmus University Medical Center, Rotterdam, Netherlands; ^3^ Department of Internal Medicine, Division of Nephrology, Erasmus University Medical Center, Rotterdam, Netherlands; ^4^ Department of Internal Medicine, Division of Endocrinology, Erasmus University Medical Center, Rotterdam, Netherlands; ^5^ Department of Immunology, Erasmus University Medical Center, Rotterdam, Netherlands

**Keywords:** serum immunoglobulins, adult human beings, determinants, systematic review, meta-analysis

## Abstract

**Background:**

An up-to-date overview of determinants of serum immunoglobulins in adults is pivotal for clinical practice and research, but currently lacking. We therefore performed a systematic review and meta-analysis to identify determinants of serum immunoglobulin levels.

**Methods:**

Embase, Web of Science, Medline, Cochrane, and Google Scholar were searched from inception to July 11^th^, 2019 for articles reporting on determinants of serum immunoglobulin A, G or M (IgA, IgG or IgM) in adult humans. Random and fixed effect models were applied to obtain pooled mean differences (MDs) and 95% confidence intervals (CIs) for the association of age and sex with serum immunoglobulins.

**Results:**

We retrieved 117 articles reporting on determinants of serum immunoglobulins, of which 28 could be meta-analyzed. Older compared to younger individuals had higher IgA (MD: 0.38; CI: 0.18 – 0.58), but lower IgM levels (MD: -0.40; 95%: -0.66 – -0.14). Men had higher IgA (MD: 0.22; CI: 0.03 – 0.42), but lower IgM levels (MD: -0.21; CI: -0.32 – -0.10) than women. Age and sex did not influence IgG. Caucasian ethnicity was associated with lower IgA, IgG, and IgM. Smoking and corticosteroid use were associated with lower IgG. Positive associations were reported of probiotics with IgG, alcohol with IgA, hypertension with IgA and IgG, and acute psychological stress with IgA, IgG, and IgM.

**Conclusions:**

Older age and male sex are associated with higher IgA, but lower IgM, and urge investigation of age- and sex-specific reference ranges of immunoglobulins. Other identified determinants were ethnicity, diet, lifestyle and cardio-metabolic factors.

## Introduction

Serum immunoglobulins are part of the adaptive immune system and comprise five classes, including immunoglobulin A, G, and M (IgA, IgG, and IgM). IgM provides a rapid immune response and is involved in tissue homeostasis, whereas IgG and IgA are long-lasting high-affinity antibodies, the latter mainly providing mucosal immunity (1). Immunoglobulin measurements are used for diagnosis and monitoring of various diseases, including primary immunodeficiencies and autoimmune diseases. Reference ranges of immunoglobulins are based on the 2.5^th^ and 97.5^th^ percentiles in healthy adults. However, several potential determinants of serum immunoglobulins, including age and sex, are not generally considered in the interpretation of immunoglobulin levels.

Aging is associated with an increased ratio of memory to naive B-cells (2), which may lead to lower IgM and higher IgA and IgG levels in older compared to younger individuals (3). Furthermore, previous population-based studies have demonstrated lower IgG (4, 5) and IgM (4), but higher IgA (4, 5) levels in men compared to women. Among others, body mass index (BMI) and lifestyle related factors such as alcohol consumption and smoking may impact serum immunoglobulin levels as well (6, 7).

However, studies performed thus far have various limitations, limiting interpretability for the general population. Most studies were cross-sectional (3–7), had a small sample size (3, 5), did not adjust for possible confounders (4, 5, 7), or had conflicting results (3, 6, 7). The last overview of factors possibly influencing serum immunoglobulin levels dates back to 1976 (8). However, this review only described a limited number of determinants, was not performed in a systematic manner, and additional literature has been published since.

In this systematic review and meta-analysis, we aim to provide an overview of determinants of serum IgA, IgG, and IgM for adequate interpretation of immunoglobulin levels in clinical practice. This could aid in defining different reference ranges for certain populations, thus changing the universal cut-off, which is currently applied to all adults. Our overview of determinants can furthermore facilitate selection of potential confounders and mediators in immunoglobulin-related research.

## Methods

This systematic review and meta-analysis was performed in accordance with the Preferred Reporting Items for Systematic Reviews and Meta-Analyses (PRISMA) guidelines. We have provided the PRISMA checklist in **Supplementary Table S1**.

### Search and Eligibility Criteria

We searched Embase, Web of Science, Medline, Cochrane, and Google Scholar from inception to July 11^th^, 2019 with the help of the Erasmus MC medical library, for articles reporting on the association between specific determinants (i.e. factors that influence) and serum levels of IgA, IgG, or IgM in adult human beings. The definition of a determinant was deliberately kept broad and encompassed a variety of factors, such as demographic features, lifestyle related factors, and interventions. No language or date restrictions were applied during the search.

We included all study types with the exception of case series, case reports, and conference papers. Relevant reviews were included to screen the reference list for potential additional articles of interest. We excluded articles performed solely in a specific patient population (e.g. studies that correlated specific diseases or treatments to serum immunoglobulin levels), genetic or family studies, studies conducted in pregnant or lactating women, and studies focusing on rare occupational exposures as determinant, as these would limit extrapolation of results to the general adult population. A detailed search strategy and full in- and exclusion criteria can be found in the **Supplementary Material**.

### Study Selection, Data Extraction, and Quality Assessment

Titles and abstracts of the retrieved articles were screened in Endnote based on predefined in- and exclusion criteria (**Supplementary Material**). Similarly, full text articles were screened and if full texts were not available, we contacted first authors. We used a predefined data extraction form to extract relevant information of included studies on study design and setting, participants, included determinants, immunoglobulin assessment and serum levels, and study quality. For cross-sectional studies an adapted version of the Newcastle Ottawa Scale was used, as previously described by Modesti et al. (9). The Cochrane risk of bias tool was implemented for (non)randomized controlled clinical trials (RCTs), whereas we used the NIH quality assessment tool for before-after studies (10, 11). Screening and extraction were performed by two independent reviewers (SRK, ACB) and discussed with a third reviewer (LC) in case of disagreement.

### Meta-Analyses

When ≥2 comparable studies assessed the association of a certain determinant with immunoglobulins, and when means and standard deviations (SDs) were provided or could be calculated and converted to grams per liter (g/l) based on the given information, we included these in subsequent meta-analyses. Both random (DerSimonian-Laird) and fixed effect models were used to pool mean differences (MDs) and 95% confidence intervals (CIs), and the random effect models were reported as main results. Pooled results were shown in forest plots, and an I^2^ statistic was calculated for heterogeneity. Publication bias was assessed through funnel plots and the Egger test. All analyses were performed in R [metacont and metafor packages, R-project, The R Foundation for Statistical Computing (2019), version 3.5.3].

### Sensitivity and Stratified Analyses

We performed predefined sensitivity analyses by excluding outliers in the funnel plots and stratification analyses by mean publication year, ethnicity, World Health Organization (WHO) region, and older vs younger age groups provided that these were possible based on the retrieved information.

## Results

### Study Selection

We identified 9742 records after removing duplicates and added 16 articles identified through the references of retrieved reviews. Of these, 226 were eligible for full text screening. Finally, 117 articles were included in the systematic review and 28 could be meta-analyzed (**Supplementary Figure S1**).

### Study Characteristics

The included 117 articles were published between 1966 and 2019, with sample sizes ranging between 2 and 12 373 and mean age ranging between 21 and 74 years. Participants were either randomly drawn from the community, blood donors, or university/hospital employees, although population source was not always reported. Most studies were cross-sectional, 18 were RCTs, and 24 were before-after studies. Included studies were performed in Europe (n=48), Asia (n=28), North-America (n=25), Australia (n=5), Africa (n=4), and South-America (n=3). Remaining studies combined geographical sites (**Table 1**). Most studies assessed the association of age or sex with serum immunoglobulin levels. Other determinants included diet, ethnicity, smoking, alcohol consumption, cardio-metabolic risk factors, and other lifestyle related factors, with most studies including multiple determinants. A summary of characteristics of the included studies is provided in **Table 1** and a complete overview including quality scores is provided in **Supplementary Table S2**.

**Table 1:** Summarized descriptive statistics of included studies.

Determinant	N studies	Design	Continents^a^	Ethnicities^a^	Range N participants	Range age participants	Included immunoglobulins
Age	41	Cross-sectional (39), before-after study (1), non-randomized controlled clinical trial (1)	North America (12), Europe (22), Asia (7), South America (1)	Caucasian (18), African (5), Asian (4), Other (3), NR (17)	20-3213	18-106	IgA (34), IgG (37), IgM (36)
Sex	36	Cross-sectional (35), before-after study (1)	North America (7), Europe (19), Asia (6), South America (3), Africa (3)	Caucasian (16), African (7), Asian (5), Other (5), NR (10)	12-3213	18-98	IgA (30), IgG (30), IgM (32)
Diet	23	Cross-sectional (3), before-after study (10), non-randomized controlled clinical trial (2), RCT (8)	North America (4), Europe (8), Asia (9), Africa (2)	Caucasian (2), Asian (8), Other (1), NR (12)	5-1291	18-90	IgA (20), IgG (21), IgM (19)
Ethnicity	18	Cross-sectional (18)	North America (4), Europe (9), Asia (5), South America (1), Africa (2), Australia (2)	Caucasian (14), African (9), Asian (6), Other (6)	30-1799	18-95	IgA (17), IgG (17), IgM (16)
Smoking	12	Cross-sectional (12)	North America (2), Europe (5), Asia (4), Australia (1)	Caucasian (3), African (1), Asian (2), Other (1), NR (6)	23-3508	18-92	IgA (8), IgG (11), IgM (9)
Hormones (endogenous and exogenous)	8	Cross-sectional (2), before-after study (3), non-randomized controlled clinical trial (1), RCT (2)	North America (3), Europe (3), South America (1), Africa (1)	Caucasian (1), African (1), Other (1), NR (5)	9-200	18-69	IgA (7), IgG (7), IgM (6)
Alcohol consumption	6	Cross-sectional (3), before-after study (2), RCT (1)	Europe (3), Asia (2), Australia (1)	Caucasian (2), Asian (2), NR (2)	5-3508	18-92	IgA (3), IgG (4), IgM (3)
Cardiometabolic risk factors	4	Cross-sectional (4)	Europe (2), Asia (2)	Caucasian (2), Asian (2)	174-12 373	18-92	IgA (3), IgG (4), IgM (4)
Other life style factors^b^	12	Cross-sectional (1), before-after study (7), RCT (4)	North America (2), Europe (2), Asia (6), South America (1), Australia (1)	Asian (3), Other (1), NR (8)	2-76	19-63	IgA (9), IgG (12), IgM (10)
Miscellaneous^c^	8	Cross-sectional (5), before-after study (2), longitudinal (1)	North America (1), Europe (6), Asia (1)	Caucasian (3), NR (4), Asian (1)	15-927	18-94	IgA (8), IgG (8), IgM (8)

a Some studies included multiple countries/ethnicities.

b Comprises relaxation techniques (n=1), aromatherapy (n=1), massage (n=1), Tai Chi (n=1), exercise (n=1), psychological stress (n=4), and sleep deprivation (n=3).

c Comprises intelligence (n=1), amoxicillin/clavulanic acid (n=1), air pollution (n=1), external temperature (n=2), and blood group (n=3).

NR, not reported; IgA, immunoglobulin A; IgG, immunoglobulin G; IgM, immunoglobulin M; RCT, randomized controlled trial.

#### Association of Age and Sex with Serum Immunoglobulins

Age was included in 41 of the identified studies (4, 6, 12–50). Studies compared means or medians with SDs/ranges between older and younger age groups, or provided a correlation coefficient/beta for the relationship between age and immunoglobulins. Studies used different cut-off values for their younger (19 to 54 years) and older (40 to >100 years) age groups. Some studies employed multiple age groups with varying intervals (4, 19, 21, 23, 25, 30, 33, 39, 40, 43, 46, 49).

Most studies reported higher serum IgA in the older compared to younger individuals (4, 6, 13–16, 20, 22, 23, 25–28, 33, 36, 39, 40, 42, 43, 45–49). Results were heterogeneous for IgG, with 19 studies not reporting an association between age and IgG (14–18, 20, 22, 26, 28, 31–34, 38, 41, 44–46, 50), and 15 studies reporting higher IgG levels (4, 6, 12, 25, 27, 30, 35–37, 40, 42, 43, 47–49) in older compared to younger individuals. Overall, no association was found between age and serum IgM (6, 14–18, 20, 25, 26, 28, 30, 31, 35, 36, 44–46, 48, 49).

Thirty-six articles assessed differences in serum immunoglobulin levels between men and women (4–6, 13, 15, 16, 18, 19, 24, 26, 28, 31, 33–35, 39, 40, 42–44, 48, 50–64). Most studies did not report an association of sex with IgA or IgG (16, 18, 26, 28, 31, 33, 35, 39, 43, 44, 48, 51–54, 56–64). Most studies reported lower IgM levels in men compared to women (6, 15, 19, 24, 28, 31, 33–35, 39, 40, 42, 43, 48, 54, 57, 60, 61).

Twenty-eight studies reporting on age and/or sex were suitable for inclusion in the meta-analysis (**Supplementary Figure S1**).

#### Association of Diet With Serum Immunoglobulins

The association of a dietary determinant with serum immunoglobulin levels was assessed in 23 studies. The majority reported on supplementation of a micro-/macronutrient or probiotic (22, 29, 65–80), and few on nutritional status or fasting in relation to immunoglobulin levels (56, 81–84).

Five studies assessed the association of probiotics with serum immunoglobulins and generally found higher immunoglobulin levels (mostly IgG) after probiotic use compared to baseline (**Supplementary Table S2**) (71–73, 78, 80). Ascorbate (vitamin C) supplementation did not affect serum IgA and IgG, however one study reported an increase in serum IgM (29, 66, 74, 77). Ramadan fasting was associated with lower IgG levels compared to the preceding month (83, 84). Most dietary components were not or positively associated with immunoglobulin levels (**Supplementary Table S2**) (22, 67–69, 79). Consumption of Lycium Barbarum juice (65), resistant corn starch (75), or saffron tablets (70) was associated with higher IgG levels, whereas saffron supplementation and roots of North American ginseng were associated with lower IgM and IgA levels respectively (70, 76). Three observational studies assessed the relation between dietary components and serum immunoglobulins (56, 81, 82) and only established a positive correlation of dietary energy and carbohydrates with IgA (81), and a negative association between 25-hydroxyvitamin D levels and IgA (82).

#### Association of Ethnicity With Serum Immunoglobulins

Eighteen studies described the influence of ethnicity on serum immunoglobulin levels (17, 28, 33, 42, 45, 47, 50, 55, 59, 63, 85–92). Caucasians had lower immunoglobulin levels than Africans, Asians, Amazonians, or Melanesians (17, 33, 42, 50, 59, 63, 86–89, 91, 92). An Afghan study reported higher immunoglobulin levels in the Hazaras compared to other tribes (85). Two studies compared immunoglobulin levels between inhabitants of large Asian (28) or European (45) cities, but did not find any differences. One study compared mean immunoglobulin levels between inhabitants of various cities throughout the world and found highest IgG and IgM levels in Nigeria, and lowest IgM levels in Mexico city (90). Two studies reported different immunoglobulin levels in various ethnic groups stratified by sex (**Supplementary Table S2**) (47, 55).

#### Association of Smoking With Serum Immunoglobulins

Twelve studies assessed the association of smoking with serum immunoglobulin levels (6, 51, 89, 93–101). The definition of smoking was self-reported and heterogeneous (**Supplementary Table S2**).

Three studies reported lower IgA levels in smokers compared to controls (98, 100) or compared to secondhand smokers (97). Five studies did not report an association of smoking with IgA (6, 51, 93, 99, 101). Seven studies reported lower IgG levels in smokers compared to non-smokers (6, 89, 94, 95, 98–100) or compared to ex-smokers (94), regardless of the number of daily cigarettes smoked and smoking duration (89). Although nicotine replacement therapy was associated with lower IgG levels than smokeless tobacco, there were no differences between these and the non-nicotine using control group (101). Three studies did not report an association between smoking and IgG (61, 93, 97). Seven studies did not find an association between smoking and serum IgM (6, 51, 93, 97, 99–101), whereas two studies reported lower IgM levels in smokers compared to non-smokers (96, 98).

#### Association of Alcohol With Serum Immunoglobulins

The relation between alcohol consumption and serum immunoglobulins was described in six studies (6, 95, 96, 102–104). A positive association was found between alcohol consumption and IgA levels (6, 102, 103). Two studies established lower IgG levels in drinkers compared to non-drinkers (6, 95), one study found no association (104), and another study reported higher IgG levels after alcohol consumption compared to abstention (103). Results for IgM were heterogeneous, with alcohol consumption being associated with lower (96) or higher (103) serum IgM, or not having an effect at all (6).

#### Association of Hormones With Serum Immunoglobulins

Eight studies assessed the association of hormones, either endogenous or exogenous (predominantly contraceptives or corticosteroids) with immunoglobulin levels (15, 53, 105–110).

The association of contraceptives with serum immunoglobulins was heterogeneous (**Supplementary Table S2**) (15, 108, 109). Menstrual phase did not affect serum immunoglobulins (53). Dehydroepiandrosterone (DHEA) did not affect immunoglobulin levels either (107). Oral corticosteroids were associated with lower IgG levels (105, 110), and a longer treatment duration led to a slower recovery of serum IgG afterwards (105). The prostaglandin E1 analog misoprostol did not change serum immunoglobulin levels (106).

#### Association of Cardio-Metabolic Risk Factors With Serum Immunoglobulins

Four studies described the association of cardio-metabolic risk factors (blood pressure and anthropometric measures such as weight and BMI) with serum immunoglobulin levels (6, 18, 56, 111). Although different definitions of high blood pressure were applied (**Supplementary Table S2**), high compared to normal blood pressure was generally associated with higher IgA and IgG levels (6, 111) and no difference in IgM levels (6, 18). Two studies reported on the association of various anthropometric measures with serum immunoglobulins (**Supplementary Table S2**) and found positive associations of obesity with IgA and IgG (6), abdominal obesity with IgA (6), and triceps skinfold thickness with IgM (56).

#### Association of Other Lifestyle Factors With Serum Immunoglobulins

Twelve studies described among others, the influence of physical activity, psychological stress, or sleep (112–123). Psychological stress, either due to blood donation (117) or a university examination (118, 119), was associated with increased levels of IgA (117–119), IgG, and IgM (117, 119). Furthermore, a positive association was established between job strain and serum IgG (120). Sleep deprivation (SD) was associated with increased serum IgA, IgG, and IgM levels in one study (121), while no differences were observed in another study (122), and serum IgA even decreased during rapid eye movement SD (REM-SD) in a third study (123). While various relaxation techniques and tai chi increased all serum immunoglobulin levels (112, 115), and an increase in IgA and IgG was seen after combined aerobic and resistance exercise respectively a full body Swedish massage (114, 116), aromatherapy did not influence serum immunoglobulins (113).

#### Association of Miscellaneous Determinants With Serum Immunoglobulins

Eight studies included determinants that could not be combined into demographic or lifestyle related groups (35, 44, 46, 58, 81, 94, 124, 125). Three studies described the influence of a hematological factor on serum immunoglobulins and found a positive association between transferrin and IgM (81), and lower IgA levels in HLA-B8 DR+ compared to HLA-B8 DR- subjects (35), while no association was found between AB0 blood group and immunoglobulins (44). Two studies investigated the influence of outside temperature and concluded that serum IgA and IgM were lowest in samples that were stored at -20C for three months compared to fresh sera or sera stored for three or four weeks (46), while sauna heat exposure increased immunoglobulin levels (58). Although mean immunoglobulin levels were comparable between inhabitants of city areas with different degrees of air pollution, IgA levels of ≥300 mg/dl (≥3.0 g/l) were more prevalent in the more polluted areas (94). Two weeks after treatment with the antibiotic amoxicillin/clavulanic acid, serum IgG was lower compared to the level two weeks before start of treatment (124). Intelligence, as measured by the Wechsler adult intelligence scale (WAIS) score was negatively associated with serum IgG, even after adjustment for age, sex, and race (125).

#### Meta-Analyses

Due to the large amount of heterogeneity in the definition of included determinants or a limited number of studies investigating a certain determinant, we could only meta-analyze results for age and sex.

##### Pooled Association of Age With Immunoglobulins

Nineteen studies reporting on age were included in the meta-analysis. We included 13 studies that reported MDs in serum immunoglobulins in older versus younger individuals (12, 14–16, 18–20, 29, 30, 32, 37, 38, 50). To ensure minimum overlap between the older and younger age groups as defined in the included studies, we employed a cut-off of 45 years. Estimates of multiple age groups were combined into overall estimates for the older and younger age groups. In addition, we separately meta-analyzed the results of six other studies that provided correlation coefficients for age and serum immunoglobulins (22, 26, 31, 35, 39, 45).

**Figure 1:**
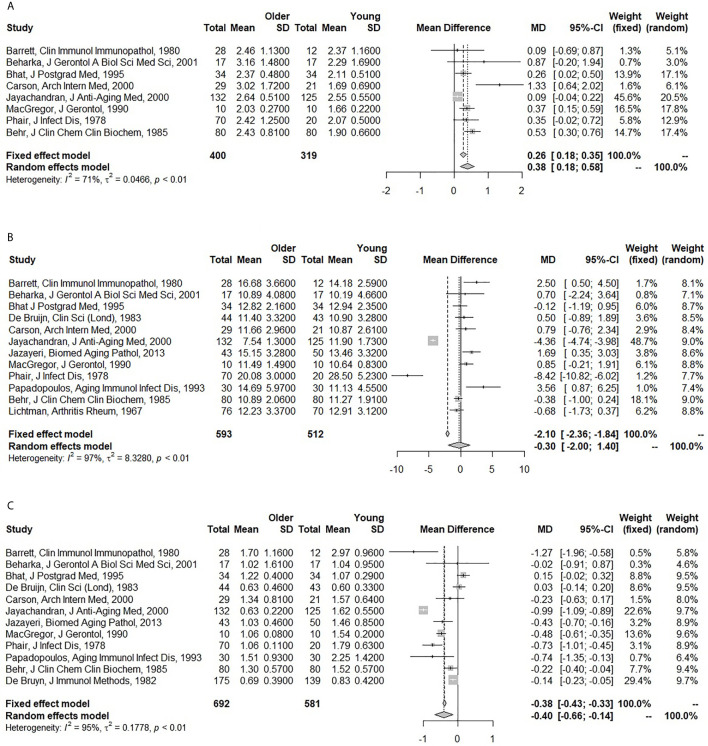
Forest plots for the association between age and serum immunoglobulin levels. **(A)** Association of age with serum immunoglobulin A (IgA) (g/l). **(B)** Association of age with serum immunoglobulin G (IgG) (g/l). **(C)** Association of age with serum immunoglobulin M (IgM) (g/l). The closed squares with horizontal lines depict the mean differences in serum immunoglobulin levels between older (≥45 years) and young (<45 years) subjects with 95% confidence intervals. The diamonds depict the pooled mean differences between the older and young age groups. The random effect model was taken as primary model.

Older individuals had higher IgA (pooled MD: 0.38; 95% CI: 0.18 – 0.58), but lower IgM levels (pooled MD: -0.40; 95% CI: -0.66 – -0.14) compared to the younger individuals. There was a trend for lower IgG levels (pooled MD: -0.30; 95% CI: -2.00 – 1.40), but this only reached significance in the fixed-effect model (pooled MD: -2.10; 95% CI: -2.36 – -1.84) (**Figure 1**). There was a substantial amount of heterogeneity for IgG (I^2^: 97%) and IgM (I^2^: 95%), and in lesser extent for IgA (I^2^: 71%) as well. The fixed-effect meta-analyses for a pooled correlation coefficient for age and serum immunoglobulins, yielded comparable results. We found a positive correlation of age with IgA, no correlation with IgG, and a negative correlation with IgM (**Supplementary Figure S2**).

##### Pooled Association of Sex With Immunoglobulins

Seventeen articles were included to obtain pooled MDs for men compared to women (5, 15, 16, 18, 19, 26, 31, 35, 50, 52–54, 57, 58, 63, 64, 103).

**Figure 2:**
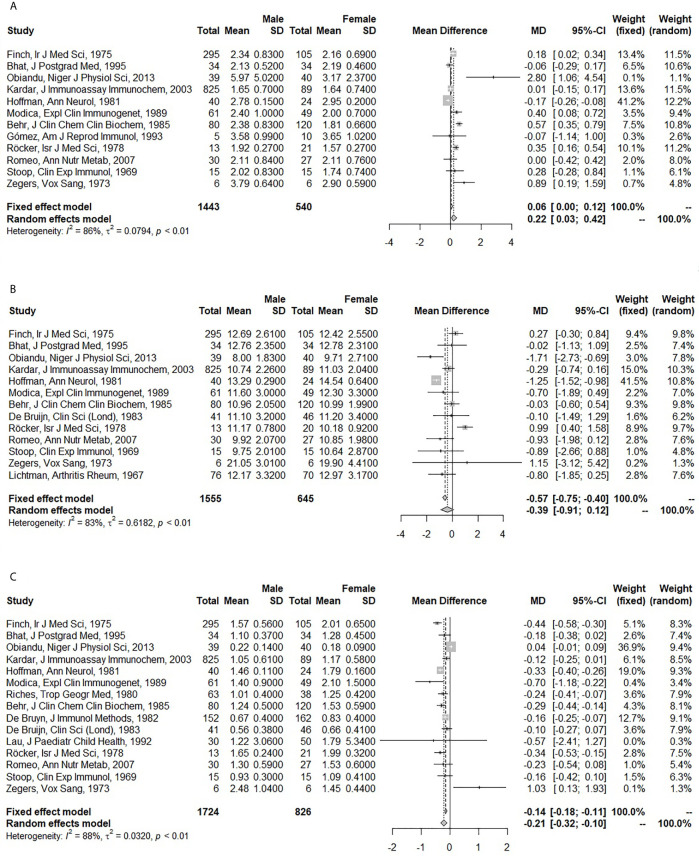
Forest plots for the association between sex and serum immunoglobulin levels. **(A)** Association of sex with serum immunoglobulin A (IgA) (g/l). **(B)** Association of sex with serum immunoglobulin G (IgG) (g/l). **(C)** Association of sex with serum immunoglobulin M (IgM) (g/l). The closed squares with horizontal lines depict the mean differences in serum immunoglobulin levels between men and women with 95% confidence intervals. The diamonds depict the pooled mean differences between men and women. The random effect model was taken as primary model.

Men had higher IgA (pooled MD: 0.22; 95% CI: 0.03 – 0.42), but lower IgM levels (pooled MD: -0.21; 95% CI: -0.32 – -0.10) than women. No difference was observed for IgG (pooled MD: -0.39; 95% CI: -0.91 – 0.12) (**Figure 2**). In the fixed effect model, the association of sex and IgA was lost (pooled MD: 0.06; 95% CI: 0.00 – 0.12), while IgG became lower in men compared to women (pooled MD: -0.57; 95% CI: -0.75 – -0.40). A large amount of heterogeneity was observed (I^2^: 86% for IgA; 83% for IgG; 88% for IgM).

#### Assessment of Publication Bias

The Egger test was significant for the association of age (P = 0.002) and sex (P = 0.013) with IgA. No statistical indications of publication bias were found for IgG and IgM. The funnel plots indicated three outliers for the association between age and IgA (15, 20, 29). For the association between age and IgG six outliers were found (12, 29, 30, 32, 37, 38), whereas for age and IgM there were seven outliers (12, 15, 16, 18, 19, 29, 38) (**Supplementary Figure S3**). In the funnel plots for sex, there were five outliers for IgA (5, 15, 16, 26, 31), four for IgG (5, 26, 52, 58), and five for IgM (5, 26, 35, 52, 63) (**Supplementary Figure S4**).

#### Sensitivity and Stratified Analyses

##### Employing a Different Cut-off for Age

Using a cut-off of 60 instead of 45 years to compare older vs younger individuals, yielded similar results and did not impact heterogeneity (**Table 2A**).

**Table 2A:** Sensitivity and stratified meta-analyses for effect of age: older *vs* younger age groups.

Immunoglobulin	N studies	Total N older (≥45y)^a^	Total N young (<45y)^a^	Fixed Effect Mean Difference (95% CI)	Random Effect Mean Difference (95% CI)	I^2^ statistic		τ^2^ statistic	
*Age ≥60y vs Age <60y*									
IgA	6	286	205	0.21 (0.11 - 0.31)	0.39 (0.11 - 0.67)	72%		0.0663	
IgG	8	359	285	-3.03 (-3.35 - -2.70)	-0.36 (-3.10 - 2.39)	97%		14.7443	
IgM	9	451	507	-0.53 (-0.59 - -0.47)	-0.56 (-0.86 - -0.25)	95%		0.1743	
*After exclusion of outliers in the funnel plot*									
IgA	5	159	93	0.33 (0.18 - 0.47)	0.33 (0.18 - 0.47)	0%		0	
IgG	6	280	265	-0.19 (-0.62 - 0.24)	-0.19 (-0.62 - 0.24)	0%		0	
IgM	5	129	128	-0.45 (-0.57 - -0.34)	-0.45 (-0.57 - -0.34)	0%		0	
*Stratified by mean publication year: published before mean*									
IgA	4	188	122	0.42 (0.28 - 0.56)	0.42 (0.28 - 0.56)	0%		0	
IgG	6	308	235	-0.27 (-0.70 - 0.16)	-0.73 (-2.39 - 0.92)	91%		3.7091	
IgM	6	407	304	-0.24 (-0.30 - -0.18)	-0.36 (-0.58 - -0.14)	89%		0.0609	
*Stratified by mean publication year: published after mean*									
IgA	4	212	197	0.17 (0.06 - 0.28)	0.44 (0.06 - 0.81)	79%		0.0921	
IgG	6	285	277	-3.18 (-3.51 - -2.85)	0.29 (-2.63 - 3.21)	97%		12.4430	
IgM	6	285	277	-0.63 (-0.71 - -0.55)	-0.39 (-0.93 - 0.15)	96%		0.4045	
*Stratified by region: North America*									
IgA	5	154	80	0.42 (0.25 - 0.60)	0.51 (0.18 - 0.84)	52%		0.0633	
IgG	6	230	150	-0.10 (-0.70 - 0.50)	-0.63 (-2.85 - 1.59)	91%		6.7568	
IgM	5	154	80	-0.52 (-0.63 - -0.40)	-0.55 (-0.81 - -0.29)	61%		0.0454	
*Stratified by region: Europe*									
IgA	1	80	80	0.53 (0.30 - 0.76)	0.53 (0.30 - 0.76)	NA		NA	
IgG	3	154	153	-0.08 (-0.63 - 0.48)	0.76 (-0.91 - 2.44)	77%		1.5743	
IgM	4	329	292	-0.13 (-0.20 - -0.06)	-0.15 (-0.30 - 0.01)	64%		0.0137	
*Stratified by region: Asia*									
IgA	2	166	159	0.13 (0.02 - 0.24)	0.15 (-0.01 - 0.30)	35%		0.0050	
IgG	3	209	209	-3.53 (-3.88 - -3.19)	-0.97 (-4.91 - 2.98)	98%		11.9020	
IgM	3	209	209	-0.65 (-0.73 - -0.56)	-0.43 (-1.21 - 0.36)	98%		0.4761	
*Stratified by ethnicity: Caucasian*								
IgA	NA	NA	NA	NA	NA	NA		NA	
IgG	2	84	79	-0.20 (-1.09 - 0.68)	-0.16 (-1.32 - 1.00)	40%		0.2853	
IgM	1	44	43	0.03 (-0.14 - 0.20)	0.03 (-0.14 - 0.20)	NA		NA	
*Stratified by ethnicity: Other*								
IgA	1	34	34	0.26 (0.02 - 0.50)	0.26 (0.02 - 0.50)	NA		NA	
IgG	3	113	118	0.32 (-0.42 - 1.05)	0.33 (-0.98 - 1.65)	67%		0.8960	
IgM	2	77	84	-0.01 (-0.15 - 0.14)	-0.13 (-0.70 - 0.44)	92%		0.1549	
*Stratified by ethnicity: NR*								
IgA	7	366	285	0.27 (0.17 - 0.36)	0.42 (0.17 - 0.66)	75%		0.0637	
IgG	8	396	315	-2.65 (-2.94 - -2.36)	-0.63 (-2.92 - 1.66)	97%		10.0099	
IgM	9	571	454	-0.48 (-0.54 - -0.43)	-0.53 (-0.83 - -0.23)	95%		0.1700	

a Unless stated other cut-off for age groups.

NA, not applicable; NR, not reported.

##### Excluding Outliers in the Funnel Plot

While excluding the outliers in the funnel plots eliminated heterogeneity, it did not affect effect estimates for the association of age with IgA, IgG, and IgM. For sex however, exclusion of the outliers led to a negative association with IgG (pooled MD: -0.32; 95% CI: -0.60 – -0.03). No differences were found for the effect of sex on IgA and IgM, although heterogeneity was greatly reduced or eliminated (**Table 2B**). We also excluded the outliers one at a time, but could not identify a particular study that explained most of the heterogeneity (data not shown).

**Table 2B:** Sensitivity and stratified meta-analyses for effect of sex: males *vs* females.

Immunoglobulin	N studies	Total N Males	Total N Females	Fixed Effect Mean Difference (95% CI)	Random Effect Mean Difference (95% CI)	I^2^ statistic	τ^2^ statistic
*After exclusion of outliers in the funnel plot*							
IgA	7	425	233	0.27 (0.16 - 0.37)	0.27 (0.14 - 0.41)	21%	0.0067
IgG	9	1168	456	-0.32 (-0.60 - -0.03)	-0.32 (-0.60 - -0.03)	0%	0
IgM	10	1283	602	-0.19 (-0.24 - -0.14)	-0.19 (-0.24 - -0.14)	0%	0
*Stratified by mean publication year: published before mean*							
IgA	6	449	291	0.06 (-0.01 - 0.13)	0.30 (0.00 - 0.60)	92%	0.1133
IgG	8	566	406	-0.59 (-0.79 - -0.39)	-0.18 (-0.97 - 0.61)	89%	0.9542
IgM	9	705	537	-0.27 (-0.31 - -0.22)	-0.25 (-0.34 - -0.16)	71%	0.0123
*Stratified by mean publication year: published after mean*							
IgA	6	994	249	0.06 (-0.06 - 0.17)	0.13 (-0.13 - 0.39)	68%	0.0575
IgG	5	989	239	-0.53 (-0.88 - -0.19)	-0.67 (-1.24 - -0.11)	48%	0.1911
IgM	6	1019	289	-0.01 (-0.05 - 0.04)	-0.15 (-0.31 - 0.01)	75%	0.0219
*Stratified by region: North America*							
IgA	1	40	24	-0.17 (-0.26 - -0.08)	-0.17 (-0.26 - -0.08)	NA	NA
IgG	2	116	94	-1.22 (-1.48 - -0.96)	-1.22 (-1.48 - -0.96)	0%	0
IgM	1	40	24	-0.33 (-0.40 - -0.26)	-0.33 (-0.40 - -0.26)	NA	NA
*Stratified by region: Europe*							
IgA	6	494	337	0.32 (0.22 - 0.41)	0.32 (0.16 - 0.48)	53%	0.0182
IgG	7	535	382	0.17 (-0.12 - 0.47)	-0.03 (-0.56 - 0.51)	62%	0.2876
IgM	9	750	583	-0.24 (-0.30 - -0.19)	-0.26 (-0.35 - -0.17)	58%	0.0103
*Stratified by region: Asia*							
IgA	2	859	123	-0.01 (-0.15 - 0.12)	-0.01 (-0.15 - 0.12)	0%	0
IgG	2	859	123	-0.25 (-0.67 - 0.17)	-0.25 (-0.67 - 0.17)	0%	0
IgM	3	889	173	-0.14 (-0.25 - -0.03)	-0.14 (-0.25 - -0.03)	0%	0
*Stratified by region: Other*							
IgA	3	50	56	0.83 (0.27 - 1.38)	1.03 (-0.22 - 2.27)	74%	0.8711
IgG	2	45	46	-1.56 (-2.55 - -0.57)	-1.06 (-3.41 - 1.28)	39%	1.5794
IgM	2	45	46	0.04 (-0.01 - 0.10)	0.43 (-0.52 - 1.38)	78%	0.3834
*Stratified by ethnicity: Caucasian*							
IgA	3	371	169	0.23 (0.09 - 0.37)	0.23 (0.09 - 0.37)	0%	0
IgG	5	449	254	-0.09 (-0.52 - 0.34)	-0.12 (-0.59 - 0.34)	6%	0.0194
IgM	5	475	253	-0.28 (-0.37 - -0.20)	-0.28 (-0.45 - -0.12)	71%	0.0238
*Stratified by ethnicity: Other*							
IgA	5	124	114	-0.13 (-0.22 - -0.05)	0.19 (-0.20 - 0.59)	80%	0.1130
IgG	5	158	135	-1.21 (-1.46 - -0.96)	-1.09 (-1.67 - -0.50)	40%	0.1692
IgM	5	149	154	-0.09 (-0.13 - -0.05)	-0.08 (-0.35 - 0.20)	95%	0.0615
*Stratified by ethnicity: NR*							
IgA	4	948	257	0.24 (0.14 - 0.34)	0.25 (-0.03 - 0.53)	84%	0.0658
IgG	4	948	256	0.05 (-0.25 - 0.34)	-0.00 (-0.71 - 0.70)	81%	0.3992
IgM	5	1100	419	-0.19 (-0.25 - -0.13)	-0.20 (-0.28 - -0.13)	30%	0.0024

NA, not applicable; NR, not reported.

##### Stratifying by Mean Publication Year

When stratified by mean publication year, the negative association between age and serum IgM was lost in the more recently published studies. The fixed effect model furthermore yielded a strong negative association between age and IgG in the more recently published studies (pooled MD: -3.18; 95% CI: -3.51 – -2.85) (**Table 2A**). For sex, stratification by mean publication year did not impact IgA. Serum IgG was lower in men compared to women in the more recently published studies (pooled MD: -0.67; 95% CI: -1.24 – -0.11), whereas the association of sex and IgM was lost in these studies (**Table 2B**).

##### Stratifying by WHO Region

Age was not associated with IgG in any WHO region. The positive association of age with IgA was lost in Asia (pooled MD: 0.15; 95% CI: -0.01 – 0.30) and the negative association of age and IgM only remained in North America (pooled MD: -0.55; 95% CI: -0.81 – -0.29) (**Table 2A**). No relation was found between sex and IgA in Asian and other regions (excluding North America and Europe). In Europe however, men compared to women had higher IgA levels (pooled MD: 0.32; 95% CI: 0.16 – 0.48). The opposite association was found in North America (pooled MD: -0.17; 95% CI: -0.26 – -0.08) although only one study was performed in that region. IgG was lower in men compared to women in the North American studies (pooled MD: -1.22; 95% CI: -1.48 – -0.96), whereas no association between sex and IgG was found in the other WHO regions. IgM was lower in men than women in all North American, European and Asian studies (pooled MDs ranging from -0.33 to -0.14). Only one study was performed in another WHO region and did not report a relation between sex and IgM (**Table 2B**).

##### Stratifying by Ethnicity

The positive association of age with IgA was not impacted by ethnicity, although most studies did not report the ethnicity of participants. While the effect estimates for the association of age with IgG and IgM were opposite in Caucasians vs subjects of other ethnicities, none of these associations reached significance. IgM levels were lower in older compared to younger subjects of no reported ethnicity (pooled MD: -0.53; 95% CI: -0.83 – -0.23) (**Table 2A**). Stratification by ethnicity only yielded an association between sex and IgA in Caucasians (pooled MD: 0.23; 95% CI: 0.09 – 0.37). Men compared to women of non-Caucasian ethnicity had lower IgG levels (pooled MD: -1.09; 95% CI: -1.67 – -0.50). Men furthermore had lower IgM levels than women, expect for the ones of non-Caucasian ethnicity (**Table 2B**).

### Summary of Identified Determinants

A graphic overview of identified determinants per immunoglobulin, both through the systematic review and meta-analyses, has been provided in **Supplementary Figure S5**.

## Discussion

In this study we have provided an up-to-date overview of published determinants of serum immunoglobulins, while also being the first to meta-analyze reported results. Age, sex, ethnicity, smoking, and psychological stress were identified as potentially important determinants. Heterogeneous and inconclusive results were found for the effect of diet, alcohol, hormones, and cardio-metabolic risk factors.

Pooled results showed 0.38 g/l higher IgA, but 0.40 g/l lower IgM levels in older compared to younger individuals. Our findings could be explained by a decline in IgM-producing B-cells at older age (3), although studies included in a recent review have shown a decline of naïve, IgM-memory, and switched-memory B-cells in the elderly (126). Increased immunoglobulin levels in elderly could indicate inflammatory disorders (e.g. Sjögren syndrome or rheumatoid arthritis) (127) or monoclonal gammopathy of undetermined significance (MGUS), an asymptomatic premalignant condition whose prevalence increases with age (128).

Furthermore, in our meta-analyses we showed 0.22 g/l higher IgA and 0.21 g/l lower IgM levels in men compared to women. This could partly be explained by hormonal differences, as testosterone application to human peripheral blood mononuclear cells led to decreased IgG and IgM production, whereas estradiol application had the opposite effect (129, 130). IgM-regulating properties of the X-chromosome were hypothesized to lead to higher levels in women, but results of family studies were inconclusive (131, 132). A recent study showed a positive effect of testosterone and a negative effect of estradiol on mucosal immunity in Amazonian adolescents, which could explain the higher IgA levels we found in men (133).

Caucasians had lower serum immunoglobulin levels than Africans, Asians, Native Americans, or Melanesians. This could be explained by environmental (lower microbial exposure) (134, 135) or genetic differences, as a study of black and white families from Richmond showed high heritability values for serum immunoglobulins, especially in white subjects (136). Furthermore, in admixed Latin-Americans, ancestry-specific single nucleotide polymorphisms regulated innate and adaptive immune responses (137). Genetic differences could also explain the higher immunoglobulin levels in Hazaras compared to other large Afghan tribes. Extensive genome analyses on worldwide human populations revealed that Hazaras were genetically more identical to Turkic populations in Central-Asia than to local populations (138). However, exploration of genetic determinants is beyond the scope of this systematic review.

The majority of included studies found that smoking was associated with lower serum IgG, and fewer studies also reported decreased IgA and IgM levels. Nicotine could stimulate the release of immunosuppressive hormones such as glucocorticoids and catecholamines (139). Furthermore, lymphocytes express nicotinic acetylcholine receptors (nAChR) and smoking reduces the expression of the α7 nAChR subunit known to regulate B-cell development, activation, and antibody production (140). A small study also indicated DNA methylation changes and upregulation of certain genes in leukocytes and lymphocytes of smokers (141).

All included studies on psychological stress reported increased immunoglobulin levels. This was expected, since these studies included acute stressors and in the initial stress response glucocorticoids and catecholamines exert immunostimulating rather than immunosuppressive effects (142). Long-term psychological stress was associated with decreased serum IgG-antibody production in mice and with decreased salivary IgA secretion in a population-based cohort of middle-aged and elderly individuals (143, 144).

We could not identify clear dietary determinants, possibly due to large heterogeneity and included studies on average having a sample size of <100 subjects and a short follow-up of a few weeks. However, most studies on probiotic use reported an increase of serum immunoglobulins. Probiotics can positively influence immune function, depending on the probiotic strain and dose and the consumer’s age (145). Studies on alcohol consumption reported an increase of IgA levels, while results for IgG and IgM were inconclusive. This could be due to a predominantly mucosal immune response, as animal studies showed alcohol-induced damage of the intestinal mucosa and disruption of the intestinal barrier function (146).

Studies reporting on the association of hypertension with serum immunoglobulins generally showed a positive association. Hypertension can activate the adaptive immune system, possibly through formation of neoantigens (147). Mouse models furthermore showed an increase in plasma cell count and serum IgG after angiotensin II administration (148). However, various immunological pathways have been described in the pathophysiology of hypertension, suggesting a bidirectional association (147, 149).

Our biggest strength lies in provision of an up-to-date qualitative (systematic review) and quantitative (meta-analysis) overview of serum immunoglobulin determinants. We were therefore able to present coherent and comparable data, indicating consistent associations of age, sex, and key environmental factors with serum immunoglobulin levels. Our results will furthermore encourage clinicians and researchers to pay close attention to factors that influence serum immunoglobulin levels in healthy adults and possibly in the context of immunosenescence. However, our study also knows some limitations. Most included studies had a moderate quality, small sample size (n <200), cross-sectional design (thus lacking longitudinal measurements of serum immunoglobulins), and were published multiple years ago (74 out of 117 included studies were published in the 20^th^ century). We were unable to draw conclusions on certain lifestyle related, cardio-metabolic, or miscellaneous determinants due to heterogeneity in definition or a limited number of studies investigating those determinants. Therefore we could only meta-analyze results for age and sex, although a fair number of articles (n=28) were included in these meta-analyses.

The results of our systematic review and meta-analysis urge investigation of age- and sex-specific reference ranges for serum IgA and IgM. Although we did not establish associations with IgG in our main analyses, quality of included studies was generally low or moderate, total number of participants was relatively low, age of the included subjects was generally restricted to the young or middle-aged adult range, and between-study variance was high, warranting further research. When interpreting immunoglobulin levels of patients, clinicians should be aware of lower IgG levels in smokers and systemic corticosteroid users, lower IgA, IgG, and IgM levels in Caucasians, and higher IgA levels in alcohol consumers. Large population-based studies are important to confirm found associations with identified determinants (especially lifestyle and cardio-metabolic factors), while taking a wide range of potential confounders into account. Furthermore, multiple age categories should be studied in order to provide robust recommendations for age-specific reference ranges of serum immunoglobulins.

## Conclusion

This systematic review and meta-analysis presents an overview of the literature highlighting determinants that influence serum immunoglobulin levels in healthy adults. In total, 117 articles published over a time span of 53 years were included. The meta-analysis indicated higher serum IgA, but lower serum IgM levels in older individuals and in males. Other identified determinants of serum IgA, IgG, and/or IgM were ethnicity, smoking, alcohol consumption, probiotics, corticosteroid use, hypertension, and acute psychological stress.

## Data Availability Statement

The original contributions presented in the study are included in the article/**Supplementary Material**. Further inquiries can be directed to the corresponding author.

## Author Contributions

SK: Conceptualization (equal), data curation (equal), formal analysis (lead), methodology (lead), validation (equal), visualization (lead), writing—original draft (lead), and writing—review and editing (equal). AB: Data curation (equal), validation (equal), and writing—review and editing (supporting). RP: Conceptualization (equal), supervision (equal), and writing—review and editing (supporting). MH: Conceptualization (equal), supervision (equal), and writing—review and editing (supporting). VD: Conceptualization (equal), funding acquisition (lead), supervision (equal), and writing—review and editing (equal). LC: Conceptualization (equal), supervision (equal), and writing—review and editing (equal). All authors contributed to the article and approved the submitted version.

## Funding

This work was supported by Takeda [grant number IIR-NLD-002671 to VD].

## Conflict of Interest

The authors declare that the research was conducted in the absence of any commercial or financial relationships that could be construed as a potential conflict of interest.
