# Supplementary material for: Determinants of Serum Immunoglobulin Levels: A Systematic Review and Meta-Analysis
Source: Front Immunol. 2021 Apr 7;12:664526. doi: 10.3389/fimmu.2021.664526 (PMC8058410; doi:10.3389/fimmu.2021.664526)
Supplement: Supplementary file 1 [file DataSheet_1.docx]

***Supplementary Material***

**Methods**

**Example of search strategy (Medline)**

((((IgA OR IgA1 OR IgA2 OR IgG OR IgG1 OR IgG2 OR IgG2a OR IgG2b OR IgG3 OR IgG4 OR IgM OR Ig-A OR Ig-A1 OR Ig-A2 OR Ig-G OR Ig-G1 OR Ig-G2 OR Ig-G2a OR Ig-G2b OR Ig-G3 OR Ig-G4 OR Ig-M) AND (serum* OR level* OR plasma* OR blood*))).ti. OR (((IgA OR IgA1 OR IgA2 OR IgG OR IgG1 OR IgG2 OR IgG2a OR IgG2b OR IgG3 OR IgG4 OR IgM OR Ig-A OR Ig-A1 OR Ig-A2 OR Ig-G OR Ig-G1 OR Ig-G2 OR Ig-G2a OR Ig-G2b OR Ig-G3 OR Ig-G4 OR Ig-M) ADJ4 (serum* OR level* OR plasma* OR blood*)) OR ((immunoglobulin* OR immunoregulator* OR globulin* OR gammaglobulin*) ADJ4 (alpha OR A OR A1 OR A2 OR G OR G1 OR G2 OR G2a OR G2b OR G3 OR G4 OR M) ADJ6 (serum* OR level* OR plasma* OR blood*)) OR ((immunoglobulin* OR immunoregulator* OR globulin* OR gammaglobulin*) ADJ3 (serum* OR level* OR plasma* OR blood*) AND (IgG OR IgA OR IgM))).ab,ti.) **AND** (Healthy Volunteers/ OR (((health* OR normal* OR non-diseas* OR general*) ADJ3 (human* OR adult* OR patient* OR person* OR subject* OR volunteer* OR population* OR participant*))).ab,ti.) *NOT (exp animals/ NOT humans/)* *NOT (news OR congres* OR abstract* OR book* OR chapter* OR dissertation abstract*).pt.*

**Inclusion criteria**

- In vivo studies describing an association between determinants and serum IgA, IgG or IgM levels
- Studies conducted in adult human beings from the general population

**Exclusion criteria**

- Case series, case reports or conference papers
- Studies performed in solely patients using immunosuppressive drugs or receiving immunoglobulins as treatment
- Studies performed solely in patients rather than in community dwelling adults
- Studies describing immunoglobulin levels in other body material than blood
- Studies focusing on immunoglobulin subclasses or specific chains or regions of immunoglobulins
- Studies focusing on immunoglobulins against pathogens or body tissue, i.e. antigen-specific immunoglobulins or auto-immunoglobulins
- Studies describing immunoglobulin levels before and after treatment for certain underlying diseases
- Studies taking immunoglobulin levels as exposure with regard to certain medical conditions as outcome
- Studies describing genes or genetic variations as determinant
- Family studies
- Studies conducted in pregnant or lactating women
- Studies on rare occupational exposures as determinant

| Supplementary Table S1. PRISMA 2009 checklist. | | | |
| --- | --- | --- | --- |
| *SECTION/TOPIC* | *#* | *CHECKLIST ITEM* | *REPORTED ON PAGE #* |
| TITLE | | | |
| Title | 1 | Identify the report as a systematic review, meta-analysis, or both. | 1 |
| ABSTRACT | | | |
| Structured summary | 2 | Provide a structured summary including, as applicable: background; objectives; data sources; study eligibility criteria, participants, and interventions; study appraisal and synthesis methods; results; limitations; conclusions and implications of key findings; systematic review registration number. | 2 |
| INTRODUCTION | | | |
| Rationale | 3 | Describe the rationale for the review in the context of what is already known. | 3 |
| Objectives | 4 | Provide an explicit statement of questions being addressed with reference to participants, interventions, comparisons, outcomes, and study design (PICOS). | 3 |
| METHODS | | | |
| Protocol and registration | 5 | Indicate if a review protocol exists, if and where it can be accessed (e.g., Web address), and, if available, provide registration information including registration number. | x |
| Eligibility criteria | 6 | Specify study characteristics (e.g., PICOS, length of follow-up) and report characteristics (e.g., years considered, language, publication status) used as criteria for eligibility, giving rationale. | 3,4, Supplementary Material |
| Information sources | 7 | Describe all information sources (e.g., databases with dates of coverage, contact with study authors to identify additional studies) in the search and date last searched. | 3,4 |
| Search | 8 | Present full electronic search strategy for at least one database, including any limits used, such that it could be repeated. | Supplementary Material |
| Study selection | 9 | State the process for selecting studies (i.e., screening, eligibility, included in systematic review, and, if applicable, included in the meta-analysis). | 4 |
| Data collection process | 10 | Describe method of data extraction from reports (e.g., piloted forms, independently, in duplicate) and any processes for obtaining and confirming data from investigators. | 4 |
| Data items | 11 | List and define all variables for which data were sought (e.g., PICOS, funding sources) and any assumptions and simplifications made. | Supplementary Table S2 |
| Risk of bias in individual studies | 12 | Describe methods used for assessing risk of bias of individual studies (including specification of whether this was done at the study or outcome level), and how this information is to be used in any data synthesis. | 4 |
| Summary measures | 13 | State the principal summary measures (e.g., risk ratio, difference in means). | 4 |
| Synthesis of results | 14 | Describe the methods of handling data and combining results of studies, if done, including measures of consistency (e.g., I^2^) for each meta-analysis. | 4 |

| Supplementary Table S1 (continued). PRISMA 2009 Checklist. | | | |
| --- | --- | --- | --- |
| *SECTION/TOPIC* | *#* | *CHECKLIST ITEM* | *REPORTED ON PAGE #* |
| METHODS | | | |
| Risk of bias across studies | 15 | Specify any assessment of risk of bias that may affect the cumulative evidence (e.g., publication bias, selective reporting within studies). | 4 |
| Additional analyses | 16 | Describe methods of additional analyses (e.g., sensitivity or subgroup analyses, meta-regression), if done, indicating which were pre-specified. | 4 |
| RESULTS | | | |
| Study selection | 17 | Give numbers of studies screened, assessed for eligibility, and included in the review, with reasons for exclusions at each stage, ideally with a flow diagram. | Supplementary Figure S1 |
| Study characteristics | 18 | For each study, present characteristics for which data were extracted (e.g., study size, PICOS, follow-up period) and provide the citations. | Supplementary Table S2 |
| Risk of bias within studies | 19 | Present data on risk of bias of each study and, if available, any outcome level assessment (see item 12). | Supplementary Table S2 |
| Results of individual studies | 20 | For all outcomes considered (benefits or harms), present, for each study: (a) simple summary data for each intervention group (b) effect estimates and confidence intervals, ideally with a forest plot. | Figure 1, Figure 2, Supplementary Figure S2 |
| Synthesis of results | 21 | Present results of each meta-analysis done, including confidence intervals and measures of consistency. | Figure 1, Figure 2, Supplementary Figure S2 |
| Risk of bias across studies | 22 | Present results of any assessment of risk of bias across studies (see Item 15). | Supplementary Figure S3, Supplementary Figure S4 |
| Additional analysis | 23 | Give results of additional analyses, if done (e.g., sensitivity or subgroup analyses, meta-regression [see Item 16]). | Table 2 |
| DISCUSSION | | | |
| Summary of evidence | 24 | Summarize the main findings including the strength of evidence for each main outcome; consider their relevance to key groups (e.g., healthcare providers, users, and policy makers). | 10,11 |
| Limitations | 25 | Discuss limitations at study and outcome level (e.g., risk of bias), and at review-level (e.g., incomplete retrieval of identified research, reporting bias). | 11 |
| Conclusions | 26 | Provide a general interpretation of the results in the context of other evidence, and implications for future research. | 11,12 |
| FUNDING | | | |
| Funding | 27 | Describe sources of funding for the systematic review and other support (e.g., supply of data); role of funders for the systematic review. | 12 |
| Moher D, Liberati A, Tetzlaff J, Altman DG, The PRISMA Group (2009). Preferred Reporting Items for Systematic Reviews and Meta-Analyses: The PRISMA Statement. PLoS Med 6(6): e1000097. doi:10.1371/journal.pmed1000097 | | | |

| **Supplementary Table S2. Results of included studies on determinants of serum immunoglobulin levels^$^** | | | | | | | |
| --- | --- | --- | --- | --- | --- | --- | --- |
| *Study* | *Determinant* | *Effect estimate* | *Results for IgA* | *Results for IgG* | *Results for IgM* | *Conclusion* | *Quality score* |
| Barrett, Clin Immunol Immunopathol, 1980 | Age | Mean (SD) in mg/dl for:   1. Aged (65-103y) vs Controls (20-35y) | 1. 246 (113) vs 237 (116) | 1. 1668 (366) vs 1418 (259)* | 1. 170 (116) vs 297 (96)** | Mean IgG levels were higher (P <0.05), whereas mean IgM levels were lower (P <0.01) in the aged. | Adapted NOS (PA Modesti): 3-0-3 |
| Bátory, Arch Gerontol Geriatr, 1984 | Age | Figures (frequency distribution patterns) in g/l presented by age groups (young = 20-40y; old = 60-98y) | IgA shows a progressive increase with aging | Low IgG levels are most frequent in the 7th decade of life | Very low or very high levels of IgM seem equally reduced with aging | IgG decreased with age, especially in the 7th decade of life, whereas IgA tended to increase with age. | Adapted NOS (PA Modesti): 3-0-2 |
| Beharka, J Gerontol A Biol Sci Med Sci, 2001 | Age | Mean (SEM) in mg/dl for:   1. Young (20-40y) vs Old (≥65y) | 1. 229 (41) vs 316 (36)*   * P <0.05 | 1. 1019 (113) vs 1089 (99) | 1. 104 (23) vs 102 (39) | Serum IgA levels increased with age, whereas serum IgG and IgM levels were similar for young and old subjects. | Adapted NOS (PA Modesti): 3-0-3 |
| Behr, J Clin Chem Clin Biochem, 1985 | Age | Mean (SD) in mg/l for:   1. Males 19-39y vs 40-60y 2. Females 19-39y vs 40-60y | 1. 2034 (689) vs 2733 (818) 2. 1767 (605) vs 2131 (689) | 1. 11020 (1680) vs 10890 (2390) 2. 11510 (2110) vs 10890 (1710) | 1. 1313 (486) vs 1157 (501) 2. 1730 (579) vs 1439 (600) | An increase with age was found for IgA. However, no P-values were provided. | Adapted NOS (PA Modesti): 2-0-2 |
| Bhat, J Postgrad Med, 1995 | Age | Mean (SD) in mg/100 ml for:   1. Age 30-45y vs 45-60y | 1. 210.93 (50.93) vs 236.53 (47.52); P<0.05 | 1. 1294.05 (235.43) vs 1281.52 (215.88); P>0.20 | 1. 107.14 (29.48) vs 122.29 (39.87); P>0.05 | Compared to individuals aged 45-60y, individuals aged 30-45y had lower IgA levels. | Adapted NOS (PA Modesti): 3-0-3 |
| Bowden, J Am Geriatr Soc, 1993 | Age | MANOVA of immunoglobulin levels by age groups (60-69, 70-79, 80-89, 90-95y) | No significant age related differences (data not shown) | No significant age related differences (data not shown) | No significant age related differences (data not shown) | No association was found between age and immunoglobulin levels. | Adapted NOS (PA Modesti): 3-0-3 |
| De Bruijn, Clin Sci (Lond), 1983 | Age | Mean (SEM) in g/l for:   1. Age 40-54y vs 55-69y | NA | 1. 10.9 (0.5) vs 11.4 (0.5) | 1. 0.60 (0.05) vs 0.63 (0.07) | No difference observed in serum IgG and IgM levels between age groups. | Adapted NOS (PA Modesti): 2-0-2 |
| De Bruyn, J Immunol Methods, 1982 | Age (by sex) | Median (range) in mg/100 ml for:   1. 20-24y male vs female 2. 25-29y male vs female 3. 30-34y male vs female 4. 35-39y male vs female 5. 40-44y male vs female 6. 45-49y male vs female 7. 50-54y male vs female 8. 55-59y male vs female 9. 60-64y male vs female 10. 65-69y male vs female 11. 70-74y male vs female 12. >75y male vs female | NA | NA | 1. 53.5 (34.0-108.8) vs 99.6 (44.9-186.3) 2. 53.6 (35.7-134.7) vs 98.5 (49.6-123.9) 3. 52.9 (14.8-144.9) vs 98.0 (32.2-121.9) 4. 67.7 (30.0-293.7) vs 59.5 (24.1-195.1) 5. 59.1 (30.8-130.5) vs 62.4 (32.1-134.2) 6. 53.2 (33.3-123.4) vs 80.4 (31.9-166.7) 7. 61.9 (28.7-130.2) vs 56.8 (27.3-207.6) 8. 41.8 (14.7-107.9) vs 51.2 (31.3-129.1) 9. 29.7 (21.0-52.7) vs 46.9 (25.6-159.4) 10. 43.6 (25.1-197.6) vs 54.2 (44.5-166.1) 11. 40.4 (19.5-149.2) vs 63.1 (29.3-229.9) 12. 41.9 (14.8-99.2) vs 45.4 (20.5-106.9) | For both sexes a decrease in IgM with age was found (P <0.02). | Adapted NOS (PA Modesti): 3-0-2 |
| Carson, Arch Intern Med, 2000 | Age | Mean (SEM) in g/l for:   1. Advanced elderly (>75y) vs Young (25-35y) | 1. 3.02 (0.32) vs 1.69 (0.15); P <.003 | 1. 11.66 (0.55) vs 10.87 (0.57); NS | 1. 1.34 (0.15) vs 1.57 (0.14); NS | Advanced elderly had higher serum IgA levels compared to the young. There were no differences in serum IgG and IgM. | Adapted NOS (PA Modesti): 3-0-3 |
| Cassidy, J Chron Dis, 1974 | Age (by sex) | Mean (range) in mg/ml for:   1. Age 20-34y Male vs Female 2. Age 25-29y Male vs Female 3. Age 30-34y Male vs Female 4. Age 35-39y Male vs Female 5. Age 40-44y Male vs Female 6. Age 45-49y Male vs Female 7. Age 50-54y Male vs Female 8. Age 55-59y Male vs Female 9. Age 60-64y Male vs Female 10. Age 65-69y Male vs Female 11. Age 70-74y Male vs Female 12. Age 75+ Male vs Female | 1. 1.32 (0.57-3.01) vs 1.28 (0.56-2.93) 2. 1.40 (0.61-3.21) vs 1.35 (0.59-3.08) 3. 1.50 (0.65-3.42) vs 1.42 (0.62-3.25) 4. 1.59 (0.70-3.65) vs 1.49 (0.65-3.42) 5. 1.70 (0.74-3.89) vs 1.57 (0.69-3.60) 6. 1.81 (0.79-4.15) vs 1.65 (0.72-3.79) 7. 1.93 (0.84-4.42) vs 1.74 (0.76-3.99) 8. 2.06 (0.90-4.72) vs 1.83 (0.80-4.20) 9. 2.20 (0.96-5.03) vs 1.93 (0.84-4.42) 10. 2.34 (1.02-5.36) vs 2.03 (0.89-4.66) 11. 2.49 (1.09-5.71) vs 2.14 (0.94-4.91) 12. 2.66 (1.16-6.09) vs 2.26 (0.98-5.16) | 1. 10.69 (6.60-17.33) vs 11.28 (6.96-18.28) 2. 10.83 (6.68-17.56) vs 11.35 (7.00-18.40) 3. 10.98 (6.77-17.79) vs 11.43 (7.05-18.52) 4. 11.12 (6.86-18.03) vs 11.50 (7.10-18.65) 5. 11.27 (6.95-18.27) vs 11.58 (7.14-18.77) 6. 11.42 (7.04-18.50) vs 11.66 (7.19-18.90) 7. 11.57 (7.14-18.75) vs 11.74 (7.24-19.02) 8. 11.72 (7.23-19.00) vs 11.81 (7.29-19.15) 9. 11.88 (7.33-19.25) vs 11.89 (7.34-19.28) 10. 12.04 (7.43-19.51) vs 11.97 (7.39-19.41) 11. 12.20 (7.52-19.77) vs 12.05 (7.44-19.54) 12. 12.36 (7.62-20.03) vs 12.13 (7.49-19.67) | NR | IgA and IgG levels tend to increase with age.  Females have higher IgG, but lower IgA levels than males.  However, no P-values for differences provided. | Adapted NOS (PA Modesti): 3-0-2 |
| Challacombe, Oral Microbiol Immunol, 1995 | Age | Figures of serum immunoglobulin levels in mg/ml by age group (20-39y; 40-59y; 60-79y; ≥80y) | Serum IgA levels did not significantly change by age | Serum IgG was significantly lower in the ≥80y group compared to the 40-59y group | Serum IgM was significantly lower in the ≥80y group compared to the 40-59y group | There was no age trend for IgA. Serum IgG and IgM levels were highest in the 40-59y group and declined in older participants, with lower values in the group ≥80y (P <0.02). | Adapted NOS (PA Modesti): 3-0-3 |
| Farges, Br J Nutr, 2012 | Age | Correlation coefficient R^2^ (P-value) with IgA in g/l | 0.013 (<0.05) | NA | NA | There was no association between IgG and IgM with age (data not shown). However, serum IgA levels increased with age | NIH assessment tool for before-after studies: 5/12 |
| Gonzalez-Quintela, Clin Exp Immunol, 2008 | Age | Description of immunoglobulin levels in mg/dl | Serum IgA levels tended to increase with age (P for trend <0.001) | Serum IgG levels tended to increase with age (P for trend <0.001) | Serum IgM showed no variation with age (P =0.54) | Serum IgA and IgG levels increase with age | Adapted NOS (PA Modesti): 2-2-3 |
| De Greef, Mech Ageing Dev, 1992 | Age (volunteers were drawn from the Senieur protocol for immunogerontological studies; sera were compared with non-senieurs that did not meet all Senieur criteria for healthy aging) | Mean (range) in mg/ml for:   1. Age 25-34y 2. Age 35-44y 3. Age 45-54y 4. Age 55-64y 5. Age 65-74y 6. Age 75-84y 7. Age >85y 8. Non-Senieur 75-84y 9. Non-Senieur >85y | 1. 1.51 (0.57-5.3) 2. 1.87 (1.40-3.7) 3. 2.17 (1.19-4.5) 4. 1.46 (0.77-2.39) 5. 1.91 (1.18-3.3) 6. 2.00 (0.67-5.9) 7. 2.34 (0.66-6.0) 8. 2.72 (0.53-7.1) 9. 3.3 (1.50-6.8) | NR | 1. 1.08 (0.58-1.68) 2. 1.33 (0.62-2.45) 3. 1.41 (0.73-5.5) 4. 1.41 (0.64-5.3) 5. 2.15 (0.94-5.7) 6. 1.31 (0.42-3.6) 7. 1.32 (0.71-2.91) 8. 0.85 (0.38-2.74) 9. 1.41 (0.38-3.9) | No P values provided, although there is a trend of increasing IgA and IgM with age. | Adapted NOS (PA Modesti): 3-0-2 |
| Grundbacher, Experientia, 1980 | Age | Description based on figure of IgM levels in IU against age per 10y (range 0-90y) | NA | NA | Each of the means for age groups 15-19, 20-29 and 30-39 is higher than for age group 10-14 (p <0.01). The IgM levels show a gradual decrease after age 40 and remain stable in women 60 years of age and older | IgM levels are highest between the ages of 15-40y, and this elevation disappears after 40. | Adapted NOS (PA Modesti): 3-0-2 |
| Haferkamp, Gerontologia, 1966 | Age | Mean in mg/ml for:   1. Age 26-45y 2. Age 46-60y 3. Age 61-91y | 1. 5.1 2. 5.4 3. 6.5 | 1. 16.1 2. 16.0 3. 23.2 | 1. 2.1 2. 2.2 3. 2.3 | IgA and IgG were higher in the older age groups, while no changes were observed for IgM (however, no P-values provided). | Adapted NOS (PA Modesti): 3-0-2 |
| Hallgren, J Immunol, 1973 | Age | Mean (SEM) in WHO potency units in relation to standard 67/95 for:   1. Age 60-69y 2. Age 70-79y 3. Age 80-89y 4. Age 90-101y | 1. 69.0 (0.0550) 2. 89.5 (0.0584) 3. 87.6 (0.0870) 4. 84.9 (0.0806)   Significant P-values:  group 1 vs 2: 0.0019  group 1 vs 3: 0.0211  group 1 vs 4: 0.0340 | 1. 72.2 (0.0452) 2. 96.5 (0.0479) 3. 92.9 (0.0715) 4. 97.7 (0.0662)   Significant P-values:  group 1 vs 2: 0.0001  group 1 vs 3: 0.0039  group 1 vs 4: 0.0005 | 1. 112.9 (0.0877) 2. 116.9 (0.0931) 3. 112.2 (0.1387) 4. 114.9 (0.1285)   Significant P-values:  none | IgA and IgG increase with age, whereas no differences were observed for IgM. | Adapted NOS (PA Modesti): 3-0-3 |
| Hoffman, Ann Neurol, 1981 | Age | Correlation coefficient (P-value) for age with immunoglobulins in mg/dl | 0.257 (<0.05) | 0.103 (P = NS) | 0.016 (P = NS) | There was a positive correlation between serum IgA and age. No such associations were found for IgG and IgM. | Adapted NOS (PA Modesti): 3-0-3 |
| Hrncir, Rev Czech Med, 1973 | Age | Correlation coefficient (p-value) for age with immunoglobulins in mg/100 ml:  1. Age in males  2. Age in females | 1. 0.1988 (<0.01) 2. 2.345 (<0.01) | 1. 0.3229 (<0.001) 2. 0.2800 (<0.005) | 1. 0.1915 (<0.05) 2. 0.1653 (>0.05) | IgA and IgG were positively associated with age in both sexes, whereas IgM was only positively associated with age in males. | Adapted NOS (PA Modesti): 3-0-3 |
| Ichihara, Clin Chem Lab Med, 2004 | Age | Beta (P-value) for association of age (adjusted for sex and city) with immunoglobulins in g/l | 0.0107 (0.0000) | 0.0029 (0.7231) | -0.0003 (0.8638) | IgA increases with increasing age. | Adapted NOS (PA Modesti): 3-2-3 |
| Jayachandran, J Anti-Aging Med, 2000 | Age | Mean (SD) in mg/ml for:   1. Aged (>60y) vs Young (20-30y) | 1. 2.64 (0.51) vs 2.55 (0.55) | 1. 7.54 (1.30) vs 11.9 (1.73)* | 1. 0.63 (0.22) vs 1.62 (0.55)* | IgG and IgM were lower in the aged (P <0.001). | Cochrane risk of bias tool: high risk |
| Jazayeri, Biomed Aging Pathol, 2013 | Age | Mean (SEM/95% CI) in g/l for:   1. Age 20-39y 2. Age 40-64y 3. Age 65+y | NR | 1. 13.46 (0.47/   12.55-14.43)   1. 13.77 (0.46/   12.85-14.70)   1. 15.15 (0.50/   14.14-16.17) | 1. 1.46 (0.12/   1.20-1.72)   1. 1.19 (0.09/   1.01-1.38)   1. 1.03 (0.07/   0.88-1.18) | IgG was significantly higher in 40-64y compared to 20-39y. | Adapted NOS (PA Modesti): 3-0-3 |
| Kalff, Clin Chim Acta, 1970 | Age | Graph with description | IgA increases with age | IgG increases with age | No significant variation with age | IgA and IgG increased with age, while IgM was unaffected. | Adapted NOS (PA Modesti): 3-0-2 |
| Kardar, J Immunoassay Immunochem, 2003 | Age | Correlation coefficient (P-value) for age with immunoglobulins in mg/dl | 0.082 (>0.05) | 0.003 (>0.05) | 0.017 (>0.05) | No correlation between age and serum IgA, IgG, and IgM levels was found. | Adapted NOS (PA Modesti): 3-0-3 |
| Lichtman, Arthritis Rheum, 1967 | Age (by sex and ethnicity) | Mean (SEM) in mg/100 ml for:  Males   1. Age 35-54 vs 55-74 White 2. Age 35-54 vs 55-74 Negro   Females   1. Age 35-54 vs 55-74 White 2. Age 35-54 vs 55-74 Negro | NR | 1. 1081 (59) vs 1094 (74) 2. 1347 (89.5)   vs 1332 (58.2)   1. 1228 (33.6) vs 1086 (60.7) 2. 1537 (63.6)   vs 1417 (96.3) | NR | IgG levels were not influenced by age. | Adapted NOS (PA Modesti): 3-0-3 |
| MacGregor, J Gerontol, 1990 | Age | Mean (SD) in mg/dl for:   1. Elderly (>75y) vs Young (up to 30y) | 1. 203 (27) vs 166 (22); P = NS | 1. 1149 (149) vs 1064 (83); P = NS | 1. 106 (8) vs 154 (20); P = 0.032 | Elderly above 75y had significantly lower serum IgM levels compared to their younger controls. | Adapted NOS (PA Modesti): 3-0-2 |
| Maddison, Bull World Health Organ, 1975 | Age, sex and race | Mean (SD)/95% CI in IU/ml for:   1. White Male 20-40y 2. White Male 41-60y 3. White Male 61-80y 4. White Female 20-40y 5. White Female 41-60y 6. White Female 61-80y 7. Black Male 20-40y 8. Black Male 41-60y 9. Black Male 61-80y 10. Black Female 20-40y 11. Black Female 41-60y 12. Black Female 61-80y   F ratio (P-value) for:   1. Age | 1. 117 (42)/59-209 2. 145 (61)/56-271 3. 157 (69)/64-294 4. 117 (51)/59-231 5. 143 (71)/44-264 6. 130 (61)/47-244 7. 147 (61)/53-274 8. 164 (87)/45-383 9. 210 (95)/82-432 10. 143 (46)/76-230 11. 178 (71)/72-310 12. 199 (70)/94-348 13. 24.32 (<0.01) | 1. 119 (25)/72-166 2. 135 (36)/78-204 3. 126 (40)/60-215 4. 131 (30)/68-183 5. 132 (33)/81-202 6. 121 (41)/68-204 7. 168 (43)/100-250 8. 192 (40)/126-270 9. 187 (39)/124-258 10. 192 (53)/112-298 11. 180 (40)/114-263 12. 187 (46)/126-282 13. 1.65 (NS) | 1. 152 (68)/58-299 2. 129 (66)/37-279 3. 144 (90)/44-321 4. 220 (95)/75-455 5. 207 (114)/60-475 6. 130 (77)/34-284 7. 169 (75)/79-321 8. 141 (69)/40-303 9. 118 (63)/44-244 10. 256 (95)/110-456 11. 152 (80)/54-333 12. 137 (108)/49-477 13. 33.24 (<0.01) | Except for white females, there was an increase (P <0.01) in mean IgA with age for both races. IgM decreased with age, this was especially seen in females of both races, and was least apparent in black males. Age did not influence IgM in white males. | Adapted NOS (PA Modesti): 3-2-3 |
| Memeo, Int J Biometeorol, 1982 | Age | Description of immunoglobulins in mg/100 ml | Maximum values of IgA were found at 30y with a slow decrease in the higher age groups. A constant increase of serum IgA levels was found in females aged 20-45y | There were no changes with advancing age | IgM levels progressively declined in males after 20y | IgA slowly decreased after 30y, whereas IgM levels declined in males after 20y. | Adapted NOS (PA Modesti): 3-0-2 |
| Modica, Expl Clin Immunogenet, 1989 | Age | Correlation coefficient for age and immunoglobulins in g/l | 0.135 (p = NS) | 0.243 (p <0.01) | -0.006 (p = NS) | IgG was positively correlated with age, whereas no relationship was found for IgA and IgM. | Adapted NOS (PA Modesti): 3-0-3 |
| Paganelli, Clin Exp Immunol, 1992 | Age | Linear regression slopes for age with immunoglobulins in mg/dl | P < 0.001 | P <0.001 | NS | IgA and IgG, but not IgM, increase with age. | Adapted NOS (PA Modesti): 3-0-3 |
| Papadopoulos, Aging Immunol Infect Dis, 1993 | Age | Mean (SEM) in mg/dl for:   1. Elderly (69-89y) vs Young (19-35y) | NA | 1. 1469 (109) vs 1113 (83); P = 0.038 | 1. 151 (17) vs 225 (26); P = 0.043 | Elderly had higher IgG, but lower IgM levels than young. | Adapted NOS (PA Modesti): 3-0-3 |
| Phair, J Infect Dis, 1978 | Age | Mean (SD) in mg/100 ml for:   1. Aged (65-88y) vs Young (27-43y) | 1. 242 (124.8) vs 207 (50); P = NS | 1. 2,008 (300) vs 2,850 (523); P = NS | 1. 106 (10.9) vs 179 (63); P <0.001 | There were no differences in mean IgA and IgG levels between aged individuals and young controls. IgM however, was lower in the aged. | Adapted NOS (PA Modesti): 3-0-3 |
| Quintiliani, Boll Ist Sieroter Milan, 1976 | Age | Mean (SD), unit not reported for:   1. Male vs Female 20-29y 2. Male vs Female 30-39y 3. Male vs Female 40-49y 4. Male vs Female 50-59y 5. Age <40 vs >40y 6. Male <40 vs >40y 7. Female <50 vs >50y   * P <0.01  Correlation coefficient (p-value) for:   1. Age 2. Age in males 3. Age in females | 1. 124 (48.1) vs 115 (39.8) 2. 119 (42.0) vs 124 (41.4) 3. 144 (47.3) vs 140 (42.6) 4. 139 (51.9) vs 130 (47.6) 5. 120 (43) vs 139 (47.5) 6. NA 7. NA   P-value for difference between age groups:  - Males 20-29y vs 40-49y: <0.05  - Males 30-39y vs 40-49y: <0.01  - Females 20-29y vs 40-49y: <0.01   1. 0.190 (<0.01) 2. 0.199 (<0.01) 3. 0.174 (<0.01) | 1. 151 (36.8) vs 148 (35.4) 2. 142 (32.0) vs 152 (44.6) 3. 138 (33.4) vs 158 (37.2)* 4. 137 (35.7) vs 151 (39.1)* 5. NA 6. NA 7. NA   P-value for difference between age groups:  - Males 20-29y vs 50-59y: <0.05   1. -0.034 (NS) 2. -0.133 (<0.01) 3. 0.083 (NS) | 1. 177 (71.5) vs 210 (75.0)* 2. 159 (69.0) vs 214 (76.2)* 3. 145 (63.2) vs 193 (85.6)* 4. 146 (59.2) vs 166 (84.8) 5. NA 6. 168 (70.7) vs 146 (61.1) 7. 206 (79.3) vs 166 (84.8)   P-value for difference between age groups:  - Males 20-29y vs 40-49y: <0.01  - Females 20-29y vs 50-59y: <0.01  - Females 30-39y vs 50-59y: <0.01  - Females 40-49y vs 50-59y: <0.05   1. -0.192 (<0.01) 2. -0.172 (<0.01) 3. -0.199 (<0.01) | There was a positive correlation for IgA and negative correlation for IgM with age. IgG was negatively correlated with age in males only. | Adapted NOS (PA Modesti): 3-0-3 |
| Radl, Clin Exp Immunol, 1975 | Age | Mean (range) in mg/ml for/P-value compared to >95y:   1. Age 20-30y 2. Age 41-50y 3. Age 51-65y 4. Age >95y | 1. 1.93 (1.18-3.23)/ <0.01 2. 1.45 (0.63-3.36)/ <0.001 3. 1.65 (0.76-3.56)/ < 0.001 4. 2.54 (1.16-5.54) | 1. 10.86 (7.20-16.37)/ <0.02 2. 11.09 (7.13-17.23)/ <0.02 3. 11.60 (7.66-17.57)/ NS 4. 12.48 (7.64-20.40) | 1. 1.06 (0.61-1.82)/ NS 2. 1.13 (0.52-2.48)/ NS 3. 1.18 (0.61-2.30)/ <0.01 4. 1.05 (0.34-3.24) | IgA and IgG levels in participants >95y were higher compared to the other age groups. | Adapted NOS (PA Modesti): 3-0-2 |
| Reen, Ir J Med Sci, 1981 | Age | Figure provided with description of immunoglobulins in g/l | NA | Mean IgG levels remained relatively constant throughout the different age groups, ranging from 10.1-10.7 | NA | IgG remained relatively constant in all age groups. | Adapted NOS (PA Modesti): 2-0-2 |
| Rowe, Clin Exp Immunol, 1968 | Age | Figures with description of immunoglobulins in mg/100 ml | IgA levels increased with age | Younger Gambian adults (17-40y) tended to have lower mean IgG levels than people >40y | IgM levels increase with age in Gambians | IgA, IgG, and IgM seem to increase with age, however no P-values are provided. | Adapted NOS (PA Modesti): 3-0-2 |
| Stoica, Med Interne, 1980 | Age (by sex) | Mean (95% CI) in IU/ml for:   1. 20-29y male vs female 2. 30-39y male vs female 3. 40-49y male vs female 4. 50-59y male vs female 5. 60-69y male vs female | 1. 119 (60-236) vs 114 (56-233) 2. 133 (62-286) vs 111 (51-240) 3. 131 (58-296) vs 137 (63-272) 4. 142 (85-238) vs 131 (70-254) 5. 157 (90-270) vs 142 (65-310) | 1. 121 (84-171) vs 124 (85-179) 2. 123 (50-193) vs 122 (84-177) 3. 130 (92-185) vs 129 (86-195) 4. 135 (89-204) vs 130 (91-184) 5. 143 (92-221) vs 129 (80-206) | 1. 143 (79-259) vs 204 (121-342) 2. 157 (81-301) vs 221 (120-406) 3. 146 (80-267) vs 177 (104-301) 4. 121 (64-227) vs 136 (74-251) 5. 148 (80-275) vs 155 (92-261) | IgA and IgG levels increased with age, however for IgG this was not significant for females. IgM increased with age for both sexes, peaked in the 4th decade, and then declined; a second increase was observed in the 7th decade. | Adapted NOS (PA Modesti): 2-2-3 |
| Toshkov, Folia Haematol, 1974 | Age | Description for association of age with immunoglobulins in IU/ml | There were no significant differences by age (data not shown) | There were no significant differences by age (data not shown) | There were no significant differences by age (data not shown) | Serum levels of IgA, IgG and IgM were not influenced by age. | Adapted NOS (PA Modesti): 3-0-2 |
| Vasson, Immun Ageing, 2013 | Age | Correlation coefficient (P-value) for age with immunoglobulins in g/l | 0.2 (0.04)  IgA serum levels were higher in subjects aged 40 years or over compared to younger individuals (P<0.05). | No significant difference (data not shown) | No significant difference (data not shown) | Age was positively correlated with IgA, with individuals above 40y of age having higher IgA levels. No such association was found for IgG and IgM. | Adapted NOS (PA Modesti): 2-0-3 |
| Veys, Clinica Chimica Acta, 1973 | Age | Mean (2.5^th^-97.5^th^ percentile) in mg/100 ml for:   1. Age 20-29y 2. Age 30-39y 3. Age 40-49y 4. Age 50-59y 5. Age 60-65y | 1. 174 (66-458) 2. 221 (79-612) 3. 232 (82-661) 4. 260 (101-666) 5. 270 (76-961) | 1. 1,260 (710-1,771) 2. 1,252 (780-2,005) 3. 1,278 (792-2,063) 4. 1,320 (761-2,287) 5. 1,313 (757-2,276) | 1. 85 (32-229) 2. 76 (28-197) 3. 79 (34-180) 4. 72 (23-220) 5. 79 (33-189) | People aged 20-29y had significantly lower IgA levels compared to the other age groups. For people aged 30-39y compared to the 50-59y group, IgA was also significantly lower. No age differences were found for IgG and IgM. | Adapted NOS (PA Modesti): 3-0-2 |
| Yodfat, J Fam Pract, 1975 | Age | Description of immunoglobulin levels in mg/100 ml and in IU | IgA levels in the age group 70-80y were significantly higher than in all other age groups, while mean levels in the group of 40-50y were higher than the group of 20-30y | No age effect was found with IgG mean levels, only 70-80y were found to be significantly higher than those found in the age group 20-30y | Subjects aged 50-60y have significantly lower IgM levels than those found in the age groups 18-20, 30-40, 40-50 and 60-70 | IgA was highest in 70-80y olds. IgA levels in 40-50y olds were also higher than those 20-30y. IgG was significantly higher in 70-80y compared to 20-30y. People aged 50-60y had the lowest IgM levels. | Adapted NOS (PA Modesti): 3-1-3 |
| Bátory, Arch Gerontol Geriatr, 1984 | Sex | Figures (frequency distribution patterns) presented by sex | Younger males had higher IgA levels than females | Younger males had higher IgG levels than females | No sex differences in serum IgM | Young males tend to have higher IgA and IgG concentrations than females. No sex differences were found for IgM. | Adapted NOS (PA Modesti): 3-0-2 |
| Behr, J Clin Chem Clin Biochem, 1985 | Sex | Mean (SD) for:   1. Male vs female 19-39y 2. Male vs female 40-60y | 1. 2034 (689) vs 1767 (605) 2. 2733 (818) vs 2131 (689) | 1. 11020 (1680) vs 11510 (2110) 2. 10890 (2390) vs 10890 (1710) | 1. 1313 (486) vs 1730 (579) 2. 1157 (501) vs 1439 (600) | IgA levels were higher for men, whereas IgG and IgM levels were higher in females (although not in all age groups). However, no P-values were provided. | Adapted NOS (PA Modesti): 2-0-2 |
| Bell, Am Rev Respir Dis, 1981 | Sex | Mean (SEM) in mg/100 ml for:   1. Females (F) vs Males (M) 2. Smoking F vs M 3. Nonsmoking F vs M | 1. 3.0 (0.4) vs 2.5 (0.4) 2. 3.0 (0.7) vs 2.6 (0.5) 3. 2.9 (0.5) vs 2.3 (0.8) | 1. 14.1 (0.8) vs 14.0 (0.06) 2. 13.9 (1.4) vs 13.9 (0.2) 3. 14.4 (1.1) vs 14.1 (1.5) | 1. 2.5 (0.3) vs 1.8 (0.2) 2. 2.5 (0.3) vs 1.7 (0.3) 3. 2.5 (0.6) vs 2.0 (0.2) | There were no differences by sex. | Adapted NOS (PA Modesti): 1-0-3 |
| Bhat, J Postgrad Med, 1995 | Sex | Mean (SD) for:   1. Male vs Female 2. Male vs female 30-44y 3. Male vs female 45-60y | 1. 213.15 (52.00) vs 219.20 (46.24); P=0.20 2. 212.51 (47.54) vs 209.35 (54.07); P >0.20 3. 230.64 (52.17) vs 242.42 (41.54); P >0.20 | 1. 1275.85 (235.26) vs 1277.69 (230.55); P>0.20 2. 1299.57 (219.08) vs 1288.52 (249.09); P >0.20 3. 1279.87 (215.39) vs 1283.18 (215.24); P >0.20 | 1. 109.64 (37.42) vs 128.29 (44.97); P>0.20 2. 109.15 (27.18) vs 105.13 (31.57); P =0.20 3. 117.82 (39.46) vs 126.78 (39.79); P >0.20 | No difference in serum immunoglobulin levels between males and females. | Adapted NOS (PA Modesti): 3-0-3 |
| De Bruijn, Clin Sci (Lond), 1983 | Sex | Mean (SEM) for:   1. Male vs Female (all age groups) 2. Male vs Female 40-54y 3. Male vs Female 55-69y | NA | 1. 11.1 (0.5) vs 11.2 (0.5) 2. 10.2 (0.6) vs 11.5 (0.9) 3. 12.0 (0.8) vs 10.8 (0.6) | 1. 0.56 (0.06) vs 0.66 (0.06) 2. 0.52 (0.06) vs 0.67 (0.08) 3. 0.59 (0.10) vs 0.65 (0.10) | No p-values provided for sex differences. | Adapted NOS (PA Modesti): 2-0-2 |
| De Bruyn, J Immunol Methods, 1982 | Sex (by age groups) | Median (ranges) for males vs females | NA | NA | See results reported under ‘age’ | Women had higher IgM values than men (P <0.01). | Adapted NOS (PA Modesti): 3-0-2 |
| Cassidy, J Chron Dis, 1974 | Sex (by age groups) | Mean (ranges) for males vs females | See results reported under ‘age’ | See results reported under ‘age’ | NR | Females have higher IgG, but lower IgA levels than males. IgM levels are higher in females than in males. However, no P-values provided. | Adapted NOS (PA Modesti): 3-0-2 |
| Finch, Ir J Med Sci, 1975 | Sex | Mean (SD) for:   1. Male vs Female | 1. 234 (83) vs 216 (69) | 1. 1269 (261) vs 1242 (255) | 1. 157 (56) vs 201 (65) | No P-value for differences provided. | Adapted NOS (PA Modesti): 3-0-2 |
| Gómez, Am J Reprod Immunol, 1993 | Sex | Mean (SD) for:   1. Men vs Women | 1. 357.77 (98.78) vs 364.76 (102.15); P = NS | NA | NA | No sex differences were observed for IgA. | Adapted NOS (PA Modesti): 1-0-3 |
| Gonzalez-Quintela, Clin Exp Immunol, 2008 | Sex | Median (2.5th-97.5th percentiles) for:   1. Male vs Female | 1. 274 (89-624) vs 228 (82-470)***   *** P <0.001 | 1. 1060 (701-1803) vs 1120 (694-1760)*   * P <0.05 | 1. 112 (40-305) vs 147 (50-398)***   *** P <0.001 | Multiple linear regression adjusted for age, sex, alcohol, smoking and metabolic syndrome showed a positive association of IgA with male sex (P <0.001). Serum IgM levels were positively associated with female sex (P <0.001). | Adapted NOS (PA Modesti): 2-2-3 |
| Grundbacher, Experientia, 1980 | Sex | Figure with description | NA | NA | Mean IgM was higher for females than males aged 5-49y (P ≤0.02). The sex difference seems to have disappeared completely in individuals 60 years of age and older. | Overall, women have higher IgM levels than males. This difference however, loses significance above 50y and disappears completely over the age of 60y. | Adapted NOS (PA Modesti): 3-0-2 |
| Hoffman, Ann Neurol, 1981 | Sex | Mean (SD) for:   1. Male vs Female   Correlation coefficient (P-value) for age for:   1. Male vs Female | 1. 278 (15) vs 295 (20) 2. 0.283 (P = NS) vs 0.229 (P = NS) | 1. 1,329 (29) vs 1,454 (64) 2. 0.102 (P = NS) vs 0.129 (P = NS) | 1. 146 (11) vs 179 (16) 2. 0.012 (P = NS) vs 0.043 (P = NS) | There were no sex differences for any of the immunoglobulins. | Adapted NOS (PA Modesti): 3-0-3 |
| Ichihara, Clin Chem Lab Med, 2004 | Sex (data drawn from subjects of Tokyo, Seoul, Shanghai, Hong Kong, Taipei, and Kuala Lumpur) | Beta (P-value) for association with sex (female vs male; adjusted for age and city of residence) | -0.1094 (0.0507) | 0.4389 (0.0135) | 0.1874 (0.0000) | IgM higher in females compared to males. | Adapted NOS (PA Modesti): 3-2-3 |
| Kalff, Clin Chim Acta, 1970 | Sex | Graph with description | No difference between men and women | No difference between men and women | Women had higher IgM levels than men (P <0.001) | No sex differences for IgA and IgG. IgM was higher in women. | Adapted NOS (PA Modesti): 3-0-2 |
| Kardar, J Immunoassay Immunochem, 2003 | Sex | Mean (SD)/Range for:   1. Male vs Female | 1. 165 (70)/72-374 vs 164 (74)/69-388; (P =0.947) | 1. 1074 (226)/631-1516 vs 1103 (204)/703-1503; (P =0.24) | 1. 105 (61)/39-282 vs 117 (58)/48-289; (P =0.038) | No sex difference was found for serum IgA and IgG. Females had higher IgM levels than males. | Adapted NOS (PA Modesti): 3-0-3 |
| Lau, J Paediatr Child Health, 1992 | Sex | Mean (95% reference range) for:   1. Male vs Female | No sex differences (data not shown) | No sex differences (data not shown) | 1. 122 (53-281) vs 179 (83-385)*   *P <0.05 | IgM levels were lower in males than in females. There were no differences for IgA and IgG (data not shown). | Adapted NOS (PA Modesti): 3-0-3 |
| Lichtman, Arthritis Rheum, 1967 | Sex (by age and ethnicity) | Mean (SEM) in mg/100 ml for:   1. Male vs female age 35-54 white 2. Male vs female age 55-74 white 3. Male vs female age 35-54 negro 4. Male vs female age 55-74 negro | NR | 1. 1081 (59) vs 1228 (33.6) 2. 1094 (74) vs 1086 (60.7) 3. 1347 (89.5) vs 1537 (63.6) 4. 1332 (58.2) vs 1417 (96.3) | NR | IgG was significantly higher in negro females compared to negro males. No sex differences were observed in whites. | Adapted NOS (PA Modesti): 3-0-3 |
| Maddison, Bull World Health Organ, 1975 | Sex (and age, race) | For mean (SD) by age, sex and race group: see results reported under ‘age’  F ratio (P-value) for:   1. Sex | See results reported under ‘age’   1. 0.98 (NS) | See results reported under ‘age’   1. 0.66 (NS) | See results reported under ‘age’   1. 36.93 (<0.01) | In the younger adults (20-40y) of both races, mean IgM values were higher (P <0.01) in females than in males. IgM decreased with age, especially in females of both races, and least in black males (and did not occur at all in white males). | Adapted NOS (PA Modesti): 3-2-3 |
| Melamed, Clin Immunol Immunopathol, 1987 | Sex (and ethnicity) | Mean (SD) for:   1. Males vs Females 2. Israeli Men vs Women 3. European Men vs Women 4. Asian Men vs Women 5. North African Men vs Women   Median (range) for:   1. Males vs Females | 1. 190 (75) vs 193 (77) 2. 179 (62) vs 192 (85) 3. 184 (78) vs 181 (74) 4. 193 (74) vs 196 (71) 5. 196 (75) vs 209 (85) 6. 178 (10-546) vs 182 (15-602) | NA | NA | There was no difference in overall IgA levels between men and women. However, within each ethnic group median IgA levels were higher in women than in men. | Adapted NOS (PA Modesti): 3-0-3 |
| Memeo, Int J Biometeorol, 1982 | Sex | Description | A constant increase of serum IgA levels was found in females aged 20-45y. In addition, IgA levels in 45y old females were about 1/3rd higher than in age matched males | Females displayed increased levels of IgG when compared to males of the same age | IgM levels progressively declined in males after 20y. Females had slightly higher IgM levels than males regardless of age | Females had higher IgG and IgM levels compared to males, and also had higher IgA levels in the age group >40y. | Adapted NOS (PA Modesti): 3-0-2 |
| Modica, Expl Clin Immunogenet, 1989 | Sex | Mean (SD) in g/l for:   1. Male vs Female | 1. 2.4 (1.0) vs 2.0 (0.7); P <0.001 | 1. 11.6 (3.0) vs 12.3 (3.3) | 1. 1.4 (0.9) vs 2.1 (1.5); P <0.005 | IgA levels were higher in males compared to females, and IgM levels were lower. | Adapted NOS (PA Modesti): 3-0-3 |
| Obiandu, Niger J Physiol Sci, 2013 | Sex | Mean (SEM)/range for:   1. Male vs Female | 1. 596.69 (80.31)/5.30-1410.00 vs 316.70 (37.54)/5.30-1410.00 (P<0.05 for Male vs female) | 1. 799.89 (29.27)/467.00-1307.60 vs 971.36 (42.80)/653.80-1868.00 (P<0.05 for Male vs female) | 1. 21.94 (2.31)/8.28-69.00 vs 18.29 (1.41)/8.28-40.02 | IgA was higher in males, whereas IgG was lower compared to females. | Adapted NOS (PA Modesti): 3-0-3 |
| Pongpaew, Arch Gerontol Geriatr, 1995 | Sex | Median (95% CI) for:   1. Male vs Female | 1. 353.6 (307.2-451.1) vs 367.2 (319.7-411.0); P =0.967 | 1. 1083.8 (995.0-1180.0) vs 1042.8 (1020.0-1116.0); P =0.489 | 1. 67.3 (65.0-72.7) vs 67.8 (64.0-72.7); P =0.870 | There were no differences in serum IgA, IgG and IgM between males and females. | Adapted NOS (PA Modesti): 2-0-3 |
| Quintiliani, Boll Ist Sieroter Milan, 1976 | Sex | Mean (SD), unit not reported for:   1. Male vs Female 2. Male vs Female 20-29y 3. Male vs Female 30-39y 4. Male vs Female 40-49y 5. Male vs Female 50-59y | 1. 131 (48.4) vs 127 (43.3) 2. 124 (48.1) vs 115 (39.8) 3. 119 (42.0) vs 124 (41.4) 4. 144 (47.3) vs 140 (42.6) 5. 139 (51.9) vs 130 (47.6) | 1. 142 (34.9) vs 152 (39.3) 2. 151 (36.8) vs 148 (35.4) 3. 142 (32.0) vs 152 (44.6) 4. 138 (33.4) vs 158 (37.2)* 5. 37 (35.7) vs 151 (39.1)*   * P <0.01 | 1. 157 (67.1) vs 199 (81.5) 2. 177 (71.5) vs 210 (75.0)* 3. 159 (69.0) vs 214 (76.2)* 4. 145 (63.2) vs 193 (85.6)* 5. 146 (59.2) vs 166 (84.8)   * P <0.01 | While there was no association between age and IgA, IgG and IgM were lower in males of certain age groups only. | Adapted NOS (PA Modesti): 3-0-3 |
| Radl, Clin Exp Immunol, 1975 | Sex | Description | NR | NR | IgM levels were higher in females aged 51-65y compared to age-matched males (P < 0.05) | In people aged 51-65y, IgM levels were higher in females compared to males. | Adapted NOS (PA Modesti): 3-0-2 |
| Riches, Trop Geogr Med, 1980 | Sex | Mean (SD) in g/l for:   1. Male vs Female | No significant differences (data not shown) | No significant differences (data not shown) | 1. 1.01 (0.40) vs 1.25 (0.42) | Females had significantly higher IgM levels than males. There were no sex differences for IgA and IgG. | Adapted NOS (PA Modesti): 3-0-2 |
| Röcker, Isr J Med Sci, 1978 | Sex | Median (25^th^-75^th^ percentile) in g/l for:   1. Male vs Female | 1. 1.83 (1.71-2.32) vs 1.60 (1.23-1.86) | 1. 11.01 (10.03-12.63) vs 9.58 (9.18-12.36) | 1. 1.70 (1.37-1.83) vs 2.09 (1.45-2.32) | There were no differences in serum Ig values between men and women. | NIH assessment tool for before-after studies: 4/12 |
| Romeo, Ann Nutr Metab, 2007 | Sex, alcohol consumption | Mean (SD) for:   1. Female vs male during abstention 2. Female vs male during beer consumption | 1. 207.8 (75.91) vs 206.0 (85.81) 2. 214.6 (77.58) vs 215.4 (82.91) | 1. 1,039 (184.9) vs 1,130 (203.0) 2. 957.7 (193.3) vs 1,027 (217.4) | 1. 148.5 (60.79) vs 157.8 (60.08) 2. 123.9 (53.65) vs 136.6 (63.41) | No P-values reported for differences between men and women. | NIH assessment tool for  before-after studies: 6/12 |
| Rowe, Clin Exp Immunol, 1968 | Sex (study performed in Gambians and British) | Figure with description | NR | NR | IgM levels were higher in female adults compared to males, and this difference was highly significant in the British population. Mean (SD) for British M 12.6 (5.4) vs F 17.9 (4.7); P <0.001 | IgM levels were higher in females, especially British. | Adapted NOS (PA Modesti): 3-0-2 |
| Shiddo, Trop Geogr Med, 1994 | Sex | Description in g/l | There were no sex differences | There were no sex differences | There were no sex differences | Men and women had comparable IgA, IgG, and IgM levels. | Adapted NOS (PA Modesti): 3-0-3 |
| Sinkov, Bull World Health Organ, 1973 | Sex | Mean (SD)/Range for:   1. Male vs Female | No difference for men and women (P >0.1; data not shown) | No difference for men and women (P >0.1; data not shown) | 1. 114.7 (51.5)/60-295 vs 147.0 (34.4)/90-220; (P <0.01) | Serum IgM levels were lower in men compared to women. | Adapted NOS (PA Modesti): 2-0-3 |
| Stoica, Med Interne, 1980 | Sex | Mean (95% CI) in IU/ml for:   1. Male vs Female   For estimates by age group: see results reported under ‘age’ | 1. 129 (64-261) vs 119 (58-244) | 1. 126 (85-187) vs 125 (87-181) | 1. 143 (77-262) vs 187 (102-344) | At all ages, the mean IgA levels were higher in male than in female subjects but this difference was statistically significant only in the age group of 30-39y. Mean IgM values were higher in females than in males, but this did not reach significance in subjects aged 50-69y. There were no sex related differences for IgG. | Adapted NOS (PA Modesti): 3-2-3 |
| Stoop, Clin Exp Immunol, 1969 | Sex | Mean (SD)/P-value in mg/100 ml for:   1. Males vs Females | 1. 202 (83) vs 174 (74); P = NS | 1. 975 (201) vs 1064 (287); P = NS | 1. 93 (30) vs 109 (41); P = NS | No sex differences for IgA, IgG, and IgM. | Adapted NOS (PA Modesti): 3-0-3 |
| Toshkov, Folia Haematol, 1974 | Sex | Mean (range) in IU/ml for:  1. Male vs Female | 1. 122.2 (80-352) vs 124.1 (80-208) | 1. 116.6 (84-256) vs 119.8 (92-208) | 1. 125.0 (90-198) vs 127.4 (90-198) | Serum levels of IgA, IgG and IgM were not influenced by sex. | Adapted NOS (PA Modesti): 3-0-2 |
| White, Ann Saudi Med, 1997 | Sex | Figure with description | No apparent difference between males and females | Females tend to have higher IgG levels than males | Mean for Male vs female:  0.97 vs 1.32 (P < 0.001) | There were no sex differences for serum IgA and IgG, although females had higher IgM levels than males. | Adapted NOS (PA Modesti): 3-0-3 |
| Zegers, Vox Sang, 1973 | Sex | Mean (SD) in mg/100 ml for:   1. Males vs Females | 1. 379 (64) vs 290 (59); P = NS | 1. 2,105 (301) vs 1,990 (441); P = NS | 1. 248 (104) vs 145 (44); P = 0.042 | There were no sex differences for IgA and IgG. IgM was higher in males. | Adapted NOS (PA Modesti): 3-0-3 |
| Zegers, Clinica Chimica Acta, 1975 | Sex | Mean (range; SD) for:   1. Male vs Female | 1. 109 (56-218; 45) vs 94 (29-185; 40) | 1. 107 (73-154; 22) vs 117 (64-179; 32) | 1. 144 (85-217; 47) vs 168 (57-300; 62) | No P-values for the difference provided. | Adapted NOS (PA Modesti): 2-0-2 |
| Aral, Mediators Inflamm, 2006 | Maras powder smoking, cigarette smoking (cigarette smokers had been smoking a pack of cigarettes, whereas Maras powder smokers had been using 2 packs of Maras powder for at least 5 years. Both Maras powder users and non-smoking controls were not exposed to passive smoking) | Mean (SD) for:   1. Smokers vs Controls 2. Maras smokers vs Controls | 1. 1.9 (0.8) vs 2.2 (0.9) 2. 2.3 (0.9) vs 2.2 (0.9) | 1. 12.9 (2.3) vs 11.7 (2.7) 2. 12.0 (3.7) vs 11.7 (2.7) | 1. 1.3 (0.7) vs 1.3 (0.5) 2. 1.2 (0.4) vs 1.3 (0.5) | No differences in serum IgA, IgG, and IgM levels between Maras smokers, cigarette smokers, and non-smoking controls. | Adapted NOS (PA Modesti): 1-0-3 |
| Bell, Am Rev Respir Dis, 1981 | Smoking status | Mean (SEM) for:   1. Smokers vs Nonsmokers 2. Smoking vs nonsmoking females 3. Smoking vs nonsmoking males | 1. 2.8 (0.4) vs 2.7 (0.4) 2. 3.0 (0.7) vs 2.9 (0.5) 3. 2.6 (0.5) vs 2.3 (0.8) | 1. 13.0 (0.7) vs 14.2 (0.9) 2. 13.9 (1.4) vs 14.4 (1.1) 3. 13.9 (0.2) vs 14.1 (1.5) | 1. 2.1 (0.2) vs 2.3 (0.3) 2. 2.5 (0.3) vs 2.5 (0.6) 3. 1.7 (0.3) vs 2.0 (0.2) | There were no differences by smoking status. | Adapted NOS (PA Modesti): 1-0-3 |
| Calapai, Inhal Toxicol, 2009 | Tobacco smoking (adjusted for age and sex) | Mean (95% CI/ P-value compared to never smokers) for:   1. Never smokers 2. Smokers ≤10 cigarettes/day 3. Smokers ≥20 cigarettes/day | 1. 2.17 (1.71-2.75/-) 2. 2.02 (1.61-2.53/NS) 3. 1.81 (1.47-2.22/NS) | 1. 13.11 (12.10-14.12/-) 2. 12.11 (10.94-13.41/.06) 3. 10.38 (9.26-11.64/<.01) | 1. 1.34 (1.04-1.73/-) 2. 1.14 (0.90-1.44/NS) 3. 1.06 (0.81-1.39/NS) | IgG was reduced in the heavy smokers group compared to never smokers. | Adapted NOS (PA Modesti): 2-2-3 |
| Ferson, Int J Cancer, 1979 | Smoking status (non-smokers never smoked at time of blood collection, whereas the smokers had smoked ≥20 cigarettes/day for at least a year at blood collection) | Mean (SD) for:   1. Smokers vs Non-smokers | 1. 134 (74) vs 196 (116); P <0.005 | 1. 621 (118) vs 803 (173); P <0.001 | 1. 109 (58) vs 115 (52); P <0.70 | Smokers had lower IgA and IgG levels than non-smokers. | Adapted NOS (PA Modesti): 1-0-3 |
| Gonzalez-Quintela, Clin Exp Immunol, 2008 | Smoking (smokers were subjects that smoked at least one cigarette a day or that had quit in the preceding year) | Median (2.5^th^-97.5^th^ percentiles):   1. Smoker vs Nonsmoker | 1. 224 (74-730) vs 250 (89-564) | 1. 995 (628-1775) vs 1110 (741-1760)***   *** P <0.001 | 1. 137 (43-428) vs 132 (46-367) | IgG was negatively associated with smoking. | Adapted NOS (PA Modesti): 2-2-3 |
| Gyllén, Respir Med, 2004 | Nicotine group comprised ex-smokers that used nicotine replacement therapy (NRT) or current users of oral moist snuff (smokeless tobacco). The control group comprised subjects that had never used nicotine or that had quit more than half a year ago. | Mean (SD)/Mean difference (95% CI) for:   1. Nicotine consumers vs Healthy controls | 1. 2.5 (1.1) vs 2.3 (0.8)/-0.21 (-0.2 - 0.6) | 1. 10.5 (2.2) vs 10.6 (2.0)/-0.13 (-0.9 - 0.7)   Within the nicotine group, subjects on NRT had lower levels of IgG compared with the smokeless tobacco subgroup (mean difference −1.6 g/l; 95% CI [−2.6; −0.6]) | 1. 1.4 (0.7) vs 1.6 (0.6)/-0.19 (-0.4 - 0.1) | No differences were seen between nicotine users and controls. Within nicotine consumers, NRT was associated with lower IgG levels compared to smokeless tobacco users. | Adapted NOS (PA Modesti): 4-0-3 |
| Van Larebeke, Int J Environ Health Res, 2003 | Smoking status (smokers were all present smokers, ex-smokers were subjects that quit <20 years ago, whereas non-smokers either never smoked or quit ≥20 years ago) | Mean (SD) for:   1. Smokers 2. Ex-smokers 3. Nonsmokers | NR | 1. 1,032 (218) 2. 1,192 (276) 3. 1,216 (306)   Smokers vs Nonsmokers (adjusted for age, sex and area of residence (P <0.001) | NR | Smokers compared to nonsmokers had lower IgG levels (adjusted for sex, age, area of residence). Ex-smokers compared to smokers had higher IgG levels. | Adapted NOS (PA Modesti): 2-2-3 |
| Liao, Genes Immun, 2012 | Smoking status (smokers were defined as participants that smoked daily for >6 months) | Mean (SD) for:   1. First stage Smokers vs Nonsmokers 2. Second stage Smokers vs Nonsmokers   Stages refer to different study populations, although both adult (20-69y) Chinese men | NA | 1. 12.57 (1.21) vs 13.62 (1.21); P <0.001 2. 11.90 (1.23) vs 12.71 (1.24); P <0.001 | NA | In both stages, smoking compared to non-smoking was associated with decreased IgG levels. | Adapted NOS (PA Modesti): 2-0-3 |
| Mahassni, Indian J Clin Biochem, 2019 | Smoking status (firsthand or secondhand) | Mean (SEM) for:   1. Firsthand vs Secondhand smokers   Mean (SEM)/range for:   1. Light smokers (≤10 cigarettes/day) 2. Moderate smokers (11-20 cigarettes/day) 3. Heavy smokers (21-30 cigarettes/day) 4. Very heavy smokers (≥31 cigarettes/day) | 1. 1.86 (0.09) vs 2.37 (0.15) (P-value =0.005) 2. 1.94 (0.13)/0.82-3.27 3. 1.77 (0.17)/0.74-3.37 4. 1.71 (0.29)/0.78-2.44 5. 1.96 (0.26)/1.28-3.13   P-value = 0.789 | 1. 11.92 (0.34) vs 12.24 (0.36) (P-value =0.513) 2. 12.01 (0.46)/5.16-14.66 3. 11.69 (0.68)/6.78-16.89 4. 12.02 (1.13) /8.84-16.14 5. 12.09 (1.09)/9.39-16.63   P-value = 0.976 | 1. 0.82 (0.06) vs 0.88 (0.06) (P-value =0.537) 2. 0.83 (0.10)/0.32-1.77 3. 0.86 (0.10)/0.22-1.85 4. 0.72 (0.09)/0.49-1.08 5. 0.79 (0.09)/0.41-1.01   P-value = 0.919 | IgA was lower in firsthand smokers compared to secondhand smokers. Number of cigarettes smoked per day did not influence serum immunoglobulin levels. | Adapted NOS (PA Modesti): 2-0-3 |
| Tollerud, J Clin Lab Anal, 1995 | Smoking status (never smokers were subjects that had smoked <100 cigarettes in their lifetime, ex-smokers had previously smoked cigarettes but had quit before study entry, and current smokers were subjects that smoked during the study) | Mean (SD) for:   1. Never vs Current smokers | NR | 1. 1426 (26) vs 1287 (24); P <0.001   Among current smokers, no consistent relationship between serum IgG levels and the intensity (cigarettes smoked per day) or duration (years) of cigarette smoking could be detected | NR | Serum IgG was higher in never vs current smokers. | Adapted NOS (PA Modesti): 2-2-3 |
| Wagner, J Hyg Epidemiol Microbiol Immunol, 1983 | Smoking status (in miners) | Graphs with description: mean immunoglobulin levels with 95% CI for smokers, nonsmokers and controls by length of work underground (≤10y, 11-20y, >21y) | In miners working ≤10 years, smoking status did not influence mean IgA. With longer employment duration however, controls exhibited a continuous increase in IgA whereas IgA decreased in smokers. More smokers compared to nonsmokers (P <0.002) and controls (P <0.05) had lower IgA levels | Mean IgG was similar for non-smokers and controls, regardless of employment duration. However, smokers had lower IgG levels that continue to decline with increasing length of work. More smokers (compared to nonsmokers and controls) had lower IgG levels (P <0.002) | Mean IgM levels in smokers and controls do not differ from each other. Nonsmokers have higher IgM levels from an employment duration of 11 years onward, and the difference reaches significance in the miners working >21y underground | Smokers compared to nonsmokers and controls had lower serum IgA and IgG levels. IgM levels were higher in nonsmokers, but only in miners that had worked underground >21y. | Adapted NOS (PA Modesti): 0-0-3 |
| Yang, PLoS One, 2012 | Smoking (smokers were defined as participants that smoked daily for >6 months) | Mean (SD) for:   1. Smoking yes vs no 1st stage 2. Smoking yes vs no 2nd stage   Stages refer to different study populations | NA | NA | 1. 1.36 (0.66) vs 1.47 (0.76); P =0.001 2. 1.26 (0.46) vs 1.30 (0.47); P =0.046 | In both groups, smoking was associated with lower serum IgM levels compared to nonsmokers. | Adapted NOS (PA Modesti): 3-0-3 |
| De Feo, J Clin Invest, 1995 | Alcohol (participants received a feeding nasogastric tube and when infusion started, drank either 150ml of mineral water or table white wine (12% alcohol). Then, 50ml of water or wine was given every 15 min, for a total amount of 750ml) | Description | NA | The plasma concentration of IgG was not different between the ethanol and water group and was not affected by meal administration (data not shown) | NA | Alcohol consumption did not affect serum IgG. | Cochrane risk of bias tool: some concerns |
| Gonzalez-Quintela, Clin Exp Immunol, 2008 | Alcohol consumption (1-140g/week was considered light, 141-280g/week moderate, and >280g/week were heavy drinkers) | Median (2.5^th^-97.5^th^ percentile) for:   1. Light drinker vs abstainer 2. Moderate drinker vs abstainer 3. Heavy drinker vs abstainer | 1. 239 (78-579) vs 231 (92-503) 2. 263 (54-733) vs 231 (92-503) 3. 318 (63-569) vs 231 (92-503)***   *** P <0.001 | 1. 1095 (742-1784) vs 1120 (680-1818) 2. 1050 (599-1625) vs 1120 (680-1818)* 3. 1075 (466-1922) vs 1120 (680-1818)   * P <0.05 | 1. 131 (48-386) vs 137 (44-375) 2. 130 (36-416) vs 137 (44-375) 3. 124 (40-509) vs 137 (44-375) | Multiple linear regression adjusted for age, sex, alcohol, smoking and metabolic syndrome showed a positive association of IgA with heavy drinking. IgG was negatively associated with moderate drinking. | Adapted NOS (PA Modesti): 2-2-3 |
| Kokavec, Ann Nutr Metab, 2006 | Alcohol consumption in a fasting condition | Description | Serum IgA was significantly raised after 20 g alcohol was consumed (after a 6h fast). | NA | NA | Under fasting conditions, consumption of 20g alcohol significantly raises serum IgA levels. | NIH assessment tool for before-after studies:  6/12 |
| Liao, Genes Immun, 2012 | Alcohol consumption (drinkers drink any beverage more than once a year, others are non-drinkers) | Mean (SD) for:   1. First stage drinkers vs nondrinkers 2. Second stage drinkers vs nondrinkers   Stages refer to 2 different (yet comparable) study populations | NA | 1. 12.97 (1.21) vs 13.75 (1.23); P <0.001 2. 12.24 (1.24) vs 12.50 (1.26); P =0.158 | NA | In the first stage participants, alcohol consumption was associated with decreased serum IgG levels. This was not found in the second stage group. | Adapted NOS (PA Modesti): 2-0-3 |
| Romeo, Ann Nutr Metab, 2007 | Alcohol consumption (by sex) | Mean (SD) for:   1. Abstinence vs Beer consumption Female 2. Abstinence vs Beer consumption Male | 1. 207.8 (75.91) vs 214.6 (77.58)* 2. 206.0 (85.81) vs 215.4 (82.91)* | 1. 1,039 (184.9) vs 1,130 (203.0)* 2. 957.7 (193.3) vs 1,027 (217.4)* | 1. 148.5 (60.79) vs 157.8 (60.08)* 2. 123.9 (53.65) vs 136.6 (63.41)* | Alcohol consumption is associated with higher IgA, IgG and IgM levels in both men and women (P <0.05). | NIH assessment tool for  before-after studies: 6/12 |
| Yang, PLoS One, 2012 | Alcohol consumption | Mean (SD) for:   1. Alcohol yes vs no 1st stage 2. Alcohol yes vs no 2nd stage   Stages refer to 2 different (yet comparable) study populations | NA | NA | 1. 1.32 (0.69) vs 1.43 (0.67); P =0.013 2. 1.24 (0.46) vs 1.29 (0.46); P =0.129 | In the first sample, alcohol consumption compared to no consumption decreased mean IgM. | Adapted NOS (PA Modesti): 3-0-3 |
| Agarwal, Hum Genet, 1976 | Ethnicity | Mean (SD)/range for:   1. Tajiks 2. Pushtoons 3. Hazaras 4. Osbeks | 1. 137.0 (57.25)/38-340 2. 140.0 (49.85)/45-286 3. 151.6 (53.95)/64-267 4. 149.0 (57.68)/75-323 | 1. 218.3 (52.6)/60-386 2. 219.1 (51.1)/82-338 3. 250.4 (66.6)/72-424 4. 199.8 (41.3)/137-273 | 1. 196.4 (76.68)/57-409 2. 194.1 (87.27)/56-505 3. 213.8 (92.83)/70-572 4. 204.0 (89.11)/102-273 | The Hazaras showed a significantly higher concentrations of serum IgA, IgG and IgM compared to the other tribes. | Adapted NOS (PA Modesti): 2-0-2 |
| Bowden, J Am Geriatr Soc, 1993 | Ethnicity | Mean (SD) for:   1. Japanese vs Americans | 1. 283 (116.9) vs 226 (129.4)*   * P <0.0001 | 1. 1685 (520.4) vs 1118 (402.0)*   * P <0.0001 | 1. 138 (66.4) vs 122 (72.8) | Serum IgA and IgG levels were higher in Japanese compared to Americans. | Adapted NOS (PA Modesti): 3-0-3 |
| Grove, P N G Med J, 1975 | Ethnicity | Mean (range)/P compared to Australians for:   1. Papua New Guineans of Kefaio village 2. Papua New Guinean inmates of Goroka prison 3. Australians | 1. 240 (100-380)/<0.02 2. 230 (120-340)/<0.05 3. 200 (50-350)/NA | 1. 3340 (1080-5600)/<0.001 2. 3240 (300-6200)/<0.001 3. 1560 (900-2200)/NA | 1. 200 (100-415)/<0.001 2. 190 (90-410)/<0.001 3. 150 (50-350)/NA | Serum IgA, IgG, and IgM levels were all elevated in both PNG groups compared to Australians. There were no differences between the two PNG groups. | Adapted NOS (PA Modesti): 2-0-3 |
| Ichihara, Clin Chem Lab Med, 2004 | Geographical region | Mean (lower limit-upper limit) for:   1. Tokyo 2. Seoul 3. Shanghai 4. Hong Kong 5. Taipei 6. Kuala Lumpur | 1. 2.06 (0.91-4.23) 2. 2.29 (1.10-4.05) 3. 2.28 (1.13-4.01) 4. 2.49 (1.47-4.83) 5. 2.45 (1.11-4.44) 6. 2.37 (1.10-4.19) | 1. 12.49 (8.17-17.39) 2. 12.92 (9.13-19.59) 3. 12.22 (9.19-18.66) 4. 13.36 (7.76-19.24) 5. 13.44 (9.83-19.00) 6. 13.66 (9.01-18.59) | 1. 0.98 (0.37-2.45) 2. 1.33 (0.61-3.11) 3. 1.19 (0.47-2.80) 4. 1.23 (0.48-2.86) 5. 1.27 (0.49-2.67) 6. 1.24 (0.53-2.75) | No P values provided for between city differences in Ig levels. | Adapted NOS (PA Modesti): 3-2-3 |
| Lichtman, Arthritis Rheum, 1967 | Ethnicity (by age and sex) | Mean (SEM) in mg/100 ml for:  Males   1. Age 35-54 White vs Negro 2. Age 55-74 White vs Negro   Females   1. Age 35-54 White vs Negro 2. Age 55-74 White vs Negro | NR | 1. 1081 (59) vs 1347 (89.5); P <.05 2. 1094 (74) vs 1332 (58.2); P <.02 3. 1228 (33.6) vs 1537 (63.6); P <.05 4. 1086 (60.7) vs 1417 (96.3); P <.01 | NR | IgG was higher in negroes compared to whites, regardless of age or sex. | Adapted NOS (PA Modesti): 3-0-3 |
| Maddison, Bull World Health Organ, 1975 | Ethnicity (by age and sex) | For mean (SD) by age, sex and race group: see earlier results  F ratio (P-value) for:   1. Race | See previous results (under “age”)   1. 55.33 (<0.01) | See previous results (under “age”)   1. 303.94 (<0.01) | See previous results (under “age”)   1. 0.07 (NS) | Mean IgA and IgG were higher in blacks than in whites. | Adapted NOS (PA Modesti): 3-2-3 |
| Melamed, Clin Immunol Immunopathol, 1987 | Ethnicity (by sex) | Mean (SD) for:   1. Israel 2. Europe 3. Asia 4. North Africa | Males   1. 179 (62) 2. 184 (78) 3. 193 (74) 4. 196 (75)   Females   1. 192 (85) 2. 181 (74) 3. 196 (71) 4. 209 (85)   Kruskal-Wallis X^2^ (p-value) for ethnic origin differences:   - Males: 8.32 (0.04) - Females: 15.1 (0.0017) | NA | NA | Differences between ethnic groups were significant for both men and women. Israeli men had lowest IgA levels, whereas IgA levels were highest in North African men. In women, IgA was lowest in Europeans and highest in North Africans. | Adapted NOS (PA Modesti): 3-0-3 |
| Rowe, Clin Exp Immunol, 1968 | Ethnicity | Figures with description | Adult Gambians showed somewhat higher levels than British adults. Young adult Gambians had similar levels compared to British adults | Mean IgG levels were much higher in Gambian than in British adults | The IgM levels in British adults were restricted to the lower part of the range found in Gambian adults | IgA levels increased with age, showing no remarkable differences between Gambians and British. IgG levels were higher in Gambians than in British. | Adapted NOS (PA Modesti): 3-0-2 |
| Rowe, Lancet, 1972 | Ethnicity/geographical location | Mean (95% CI) in IU/ml for:   1. Algiers, Algeria 2. Perth, Australia 3. Santiago, Chile 4. Birmingham, England 5. Offenbach, Germany 6. Osaka, Japan 7. Mexico-City, Mexico 8. Utrecht, Netherlands 9. Ibadan, Nigeria 10. Uppsala, Sweden 11. Lausanne, Switzerland | 1. 164 (84-317) 2. 127 (56-286) 3. 163 (73-365) 4. 115 (46-289) 5. 108 (48-244) 6. 129 (70-237) 7. 97 (29-327) 8. 94 (40-223) 9. 80 (31-207) 10. 126 (57-282) 11. 136 (56-334) | 1. 143 (97-213) 2. 143 (94-219) 3. 156 (83-292) 4. 123 (73-207) 5. 124 (86-178) 6. 146 (102-210) 7. 127 (82-196) 8. 116 (65-206) 9. 287 (146-567) 10. 126 (90-177) 11. 135 (87-208) | 1. 190 (84-429) 2. 191 (86-425) 3. 158 (109-228) 4. 133 (47-372) 5. 133 (59-298) 6. 144 (68-308) 7. 63 (12-333) 8. 127 (48-334) 9. 211 (34-1413) 10. 135 (52-345) 11. 176 (81-380) | Mean IgG and IgM were highest in Nigeria. Levels of IgA, and especially of IgM, were low in Mexico City. However, no P-values for the differences were provided. | Adapted NOS (PA Modesti): 2-0-2 |
| Shiddo, Trop Geogr Med, 1994 | Ethnicity | Median (2.5th- 97.5th percentile) for:   1. Healthy Mogadishu residents ≥18y 2. Healthy Swedes (military recruits) ≥18y 3. Healthy reference Swedes ≥18y 4. Healthy Swedish blood donors ≥18y | 1. 2.2 (0.6-5.8) 2. 1.5 (0.7-2.7) 3. NR (0.7-3.2) 4. 2.9 (1.0-6.6)   P-value for difference between:   - 1 and 2: <0.001 - 1 and 3: <0.002 - 1 and 4: NS | 1. 13.0 (5.0-23.0) 2. 10.0 (6.0-16.0) 3. NR (7.1-15.2) 4. 13.9 (9.8-19.0)   P-value for difference between:   - 1 and 2: <0.001 - 1 and 3: <0.001 - 1 and 4: 0.05 | 1. 1.9 (0.5-4.6) 2. 1.1 (0.6-2.0) 3. NR (0.3-1.2) 4. 1.7 (0.8-2.8)   P-value for difference between:   - 1 and 2: <0.001 - 1 and 3: <0.001 - 1 and 4: <0.001 | The Mogadishu adults had higher serum IgA and IgG levels than Swedish military recruits and reference adults. IgM levels were higher in the Mogadishu compared to the Swedish military recruits, reference adults and Gothenburg blood donors. | Adapted NOS (PA Modesti): 3-0-3 |
| Tollerud, J Clin Lab Anal, 1995 | Ethnicity | Mean (SD) for:   1. Black vs White | No significant black/white differences (data not shown) | 1. 1587 (35) vs 1209 (16)* | No significant black/white differences (data not shown) | Serum IgG was higher in black than white individuals. | Adapted NOS (PA Modesti): 2-2-3 |
| Turner, J Trop Med Hyg, 1966 | Ethnicity | Figure with description | No apparent differences between Nigerians and British | IgG had a Gaussian distribution in the British, while in the Nigerians it was non-Gaussian and broadly distributed, with higher values than the British | IgM was higher in the Nigerians | IgA was similar between British and Nigerians, IgG and IgM were higher in the Nigerians. | Adapted NOS (PA Modesti): 3-0-2 |
| Vasson, Immun Ageing, 2013 | Geographical region (as proxy for ethnicity) | Mean (SEM) for:   1. France 2. Austria 3. Spain | 1. 2.60 (0.10) 2. 2.20 (0.10) 3. 2.72 (0.12) | 1. 11.1 (0.2) 2. 11.0 (0.2) 3. 11.1 (0.3) | 1. 1.20 (0.06) 2. 1.00 (0.04) 3. 1.18 (0.07) | There were no differences in serum immunoglobulins between the countries. | Adapted NOS (PA Modesti): 2-0-3 |
| Wang, Eur J Cancer, 1977 | Ethnicity | Log10 mean (SD) for:   1. British vs Japanese | 1. 2.10 (0.21) vs 2.16 (0.15); P =NS | 1. 2.13 (0.11) vs 2.26 (0.11); P <0.01 | 1. 2.09 (0.23) vs 2.26 (0.17); P <0.001 | There were no differences in mean IgA between British and Japanese women. However, IgG and IgM were higher in Japanese compared to British women. | Adapted NOS (PA Modesti): 3-0-2 |
| Wells, Clin Exp Immunol, 1968 | Ethnicity | Mean (range) in mg/100 ml for:   1. Caucasians vs Watuts (New Guineans) | 1. 130 (52-268) vs 146 (86-300);   P = 0.15-0.20 | 1. 1023 (515-1560) vs 1919 (1370-2640); P <.001 | 1. 123 (38-220) vs 478 (87-1650); P <.0025 | IgG and IgM were higher in New Guinea natives compared to Caucasians (Australians). | Adapted NOS (PA Modesti): 3-0-3 |
| Wright, Int J Lepr Other Mycobact Dis, 1985 | Ethnicity | Mean (SD) for:   1. Dutch vs Vietnamese | 1. 148 (90) vs 187 (71); P <0.025 | 1. 162 (35) vs 256 (47); P <0.001 | 1. 239 (86) vs 427 (157); P <0.001 | Serum IgA, IgG and IgM levels were higher in Vietnamese subjects compared to Dutch. | Adapted NOS (PA Modesti): 2-0-3 |
| Yodfat, J Fam Pract, 1975 | Ethnicity (by sex) | Mean (SD/range) in mg/100ml / mean in IU for:   1. Ashkenazi 2. Kurd 3. Yemenite 4. Cochin 5. North African | Males   1. 252 (67/140-460)/ 125 2. 205 (66/110-350)/ 102 3. 161 (66/80-340)/ 80 4. 250 (112/120-560)/ 124 5. 243 (148/100-620)/ 120   Females   1. 220 (64/140-560)/ 109 2. 212 (100/100-550)/ 105 3. 187 (69/120-340)/ 92 4. 278 (157/110-650)/ 138 5. 233 (95/100-450)/ 115 | Males   1. 1,002 (376/600-2,275)/ 95 2. 1,239 (318/675-1,600)/ 129 3. 1,103 (474/700-1,875)/ 107 4. 1,130 (328/550-1,825)/ 109 5. 1,230 (450/650-2,500)/ 118   Females   1. 1,079 (217/650-1,600)/ 109 2. 1,066 (322/500-2,100)/ 103 3. 1,105 (380/625-2,000)/ 107 4. 1,422 (422/500-2,425)/ 138 5. 1,231 (438/760-2,175)/ 118 | Males   1. 145 (37/90-275)/ 170 2. 94 (33/70-180)/ 110 3. 127 (50/85-270)/ 149 4. 117 (56/75-362)/ 138 5. 109 (40/70-225)/ 128   Females   1. 145 (55/80-300)/ 170 2. 120 (36/75-150)/ 140 3. 139 (67/62-290)/ 157 4. 140 (56/62-225)/ 165 5. 128 (50/90-280)/ 150 | Both Cochins and North Africans have significantly higher mean levels of IgG than Kurds, Yemenites and Ashkenazis. Yemenites had the lowest mean IgA levels. Cochins have higher IgA levels than Kurds, Yemenites and Ashkenazis. Ashkenazis have the highest mean IgM levels. Kurds have lower IgM levels than Ashkenazis, Cochins and Yemenites. | Adapted NOS (PA Modesti): 3-1-3 |
| Zegers, Vox Sang, 1973 | Ethnicity | Figures with description (mg/100 ml) | IgA was higher in Trio/Wajana Indians compared to Dutch regardless of sex | IgG was higher in Trio/Wajana Indians compared to Dutch regardless of sex | IgM was higher in Trio/Wajana Indians compared to Dutch regardless of sex | All immunoglobulins were higher in the Indians than Dutch. | Adapted NOS (PA Modesti): 3-0-3 |
| Green, Biofeedback Self Regul, 1988 | Relaxation techniques  (subjects were divided into 2 groups that began practicing relaxation techniques at different time points for 3 weeks); sensitivity analysis done by loneliness; 11 subjects of group 1 also separately analyzed due to total duration of 6 weeks; relaxation techniques were either performed sitting or lying down for 20 minutes daily | Mean (SD) for:   1. 22nd day vs 1st day (end of 3 week relaxation period vs baseline) | 1. 140.35 (34.71) vs 131.45 (34.57)   Increase from day 1-22 was 6.77% for IgA (P <.001)  Increase for 11 subjects of group 1 over 43 day practice period: F(2,20) =3.51; P <.05 | 1. 1,246.02 (150.98) vs 1,172.42 (164.30)   Increase from day 1-22 was 6.28% for IgG (P <.001)  Increase for 11 subjects of group 1 over 43 day practice period: F(2,20) =5.81; P <.01  Mean IgG on day 1 vs day 22 for  - Low loneliness group: 110.77 vs 120.04  - High loneliness group: 112.85 vs 116.40 | 1. 169.74 (55.54) vs 158.65 (51.50)   Increase from day 1-22 was 6.99% for IgM (P <.05)  Increase for 11 subjects of group 1 over 43 day practice period: F(2,20) =17.30; P <.01 | Three or six weeks of practicing relaxation techniques increased serum IgA, IgG, and IgM. Furthermore, subjects scoring low on a loneliness scale had a larger increase in IgG over the 3-week period. | NIH assessment tool for before-after studies: 6/12 |
| Lee, Eur J Integr Med, 2017 | Aromatherapy (60 Korean females were equally divided between the aromatherapy group inhaling an essential oil blend at night for 4 weeks and a control group) | Mean (SD) for:   1. Aromatherapy vs Control Baseline 2. Aromatherapy vs Control after 2 weeks 3. Aromatherapy vs Control after 4 weeks | NA | 1. 1229.27 (141.28) vs 1249.60 (179.49); P =.628 2. 1263.33 (169.12) vs 1281.50 (183.86); P =.692 3. 1325.80 (204.23) vs 1353.70 (205.59); P =.600 | NA | There were no differences in IgG levels between the aromatherapy and control groups throughout the study. | Cochrane risk of bias tool: some concerns |
| Lovas, J Bodywork Mov Ther, 2002 | Massage (study had an ABAB design):  A = baseline with assessment only;  B = experiment with one hour full body Swedish massage + assessment | Median for:   1. A1 subject 1 vs 2 2. B1 subject 1 vs 2 3. A2 subject 1 vs 2 4. B2 subject 1 vs 2 | 1. 2.1 vs 0.86 2. 2.155 vs 0.87 3. 2.25 vs 0.9 4. 2.305 vs 0.9   Serum IgA did not significantly increase for either subject during the massage treatment phase | 1. 11.5 vs 9.4 2. 11.95 vs 10.15 3. 11.6 vs 9.9 4. 11.9 vs 10.35   Serum levels of IgG increased significantly during the treatment phases (P =0.015) | 1. 0.91 vs 1.14 2. 1.02 vs 1.165 3. 0.96 vs 1.18 4. 0.99 vs 1.14   Serum levels of IgM demonstrated a consistent trend towards significance (P =0.06) | IgG levels were significantly increased during massage treatment periods. No changes were seen in serum IgA and IgM. | NIH assessment tool for  before-after studies: 6/12 |
| Niu, Afr J Tradit Complement Altern Med, 2016 | Tai Chi (Tai chi group performed tai chi for one hour every day, whereas the control group did not receive an intervention) | Mean (SEM) for:   1. Tai chi 2 months vs control 2. Tai chi 4 months vs control 3. Tai chi 6 months vs control | 1. 4.16 (0.18) vs 3.27 (0.13); P <0.05 2. 4.42 (0.21) vs 3.27 (0.13); P <0.01 3. 4.85 (0.25) vs 3.27 (0.13); P <0.01 | 1. 13.25 (0.78) vs 11.49 (0.95); P >0.05 2. 14.83 (0.88) vs 11.49 (0.95); P <0.05 3. 15.49 (1.27) vs 11.49 (0.95); P <0.01 | 1. 1.83 (0.08) vs 1.17 (0.09); P <0.05 2. 2.41 (0.14) vs 1.17 (0.09); P <0.01 3. 2.96 (0.18) vs 1.17 (0.09); P <0.01 | Serum IgA, IgG and IgM levels increased in the tai chi group with increasing time compared to the control group. | Cochrane risk of bias tool: high risk |
| Park, J Immunol Res, 2015 | Combined aerobic and resistance exercise (10 min warming up, 40 min aerobic exercise, 30 min resistance exercise, 10 min cooling down) 3 times a week vs control | Mean (SD) for:   1. Exercise vs Control at baseline 2. Exercise vs Control after 12 weeks 3. Exercise after 12 weeks vs baseline 4. Control after 12 weeks vs baseline | 1. 211.70 (18.13) vs 217.80 (34.24) 2. 256.50 (48.75) vs 200.80 (26.55) 3. 256.50 (48.75) vs 211.70 (18.13)** 4. 200.80 (26.55) vs 217.80 (34.24)   ** P <0.01  P for interaction between groups and period was also <0.01 | 1. 1365.10 (83.46) vs 1353.40 (89.20) 2. 1376.80 (70.30) vs 1320.00 (76.47) 3. 1376.80 (70.30) vs 1365.10 (83.46) 4. 1320.00 (76.47) vs 1353.40 (89.20) | 1. 109.35 (20.98) vs 109.60 (17.35) 2. 112.78 (22.00) vs 104.00 (16.67) 3. 112.78 (22.00) vs 109.35 (20.98) 4. 104.00 (16.67) vs 109.60 (17.35) | IgA (P <0.01) and IgG (P <0.05) increased after the exercise program. Moreover, IgA levels changed significantly over time and between groups. | Cochrane risk of bias tool: some concerns |
| Al-Hazimi, Ann Saudi Med, 2004 | Psychological stress (blood donation) | Mean (SD); P value for:   1. Pre-stress vs Post-stress | 1. 2.1 (0.8) vs 2.3 (0.8); 0.0001 | 1. 13 (2.7) vs 13.7 (2.8); 0.0001 | 1. 1.1 (0.5) vs 1.3 (0.5); 0.0001 | Mean values of IgA, IgG and IgM increased after blood donation. | NIH assessment tool for  before-after studies: 5/12 |
| Kiecolt-Glaser, Psychosom Med, 1984 | Psychological stress (examination): 1^st^ sample was  obtained 1 month after a major examination and one month prior to the final examinations; 2^nd^ sample was obtained on the first day of the final exam week, after students had completed their first two examinations | Description for change in immunoglobulin levels between 2 samples | IgA increases from the first to the second sample (F=6.05; P<0.02)  Females had lower levels on both blood samples than males (F=6.39; P<0.02)  Mean increase for males: from 177.09 to 258.83  Mean increase for females: from 148.33 to 173.42 | Mean increase from 1261.44 to 1404.61 (F=1.43; P=NS) | Mean increase from 162.95 to 188.43 (F=2.61; P=NS) | A stress situation (examination) increased serum IgA levels (P<0.02). | NIH assessment tool for before-after studies: 4/12 |
| Maes, Psychoneuroendocrinology, 1997 | Psychological stress (oral examination),  serum cortisol, hematocrit | Mean (SD) for:   1. Students with high (H) vs low (L) stress perception (based on PSS); Mean (SD) provided weeks before (PRE), a day before (STRESS) and weeks after (POST) examination   Pearson correlation coefficient (P-value) for:   1. Perceived Stress Scale (PSS) score 2. State-Trait Anxiety Inventory (STAI) score 3. Serum cortisol 4. Hematocrit | 1. H PRE 203 (56), H STRESS 219 (66), H POST 204 (53); L PRE 170 (66), L STRESS 174 (71), L POST 170 (66) 2. 0.46 (0.003) 3. 0.31 (0.04) 4. 0.14 (0.2) 5. 0.63 (<0.0001)   In the STRESS condition, serum IgA was higher in students with high-stress versus low-stress perception (P = .04). In students with high stress perception, there were furthermore differences in IgA between the PRE/POST and STRESS conditions (P < 10^-4) | 1. H PRE 1055 (148), H STRESS 1145 (175), H POST 1077 (140); L PRE 1046 (175), L STRESS 1058 (185), L POST 1049 (180) 2. 0.51 (0.0009) 3. 0.40 (0.009) 4. 0.09 (0.6) 5. 0.44 (0.0002)   In students with high stress perception, there were differences in serum IgG between the PRE/POST and STRESS conditions (P < 10^-4) | 1. H PRE 102 (39), H STRESS 116 (52), H POST 109 (47); L PRE 107 (16), L STRESS 110 (43), L POST 108 (41) 2. 0.51 (0.0008) 3. 0.38 (0.01) 4. 0.07 (0.6) 5. 0.42 (0.0003)   In students with high stress perception, there were differences in serum IgM between the PRE/POST and STRESS (P = .001) | In students with high stress perception, serum IgA, IgG and IgM were higher during stress compared to the pre and post values. There were also positive correlations between the PSS score, STAI score, hematocrit and all immunoglobulins. | NIH assessment tool for before-after studies: 6/12 |
| Theorell, Psychosom Med, 1990 | Job strain, social support (measured with questionnaires); Job strain comprised demands and decision latitude. Social support comprised attachment and social integration and was graded on availability and adequacy | Mean log (SD) for:   1. Min job strain 2. Next to min job strain 3. Next to max job strain 4. Max job strain   Mean log (SD) for:   1. Min strain - Low support 2. Min strain - Intermediate support 3. Min strain - High support 4. Next to min strain - Low support 5. Next to min strain - Intermediate support 6. Next to min strain - High support 7. Next to max strain - Low support 8. Next to max strain - Intermediate support 9. Next to max strain - High support 10. Max strain - Low support 11. Max strain - Intermediate support 12. Max strain - High support | NA | 1. 2.95 (0.12) 2. 2.96 (0.11) 3. 2.98 (0.11) 4. 2.99 (0.11)   ANOVA F =9.60; P =0.05   1. 3.00 (0.18) 2. 2.96 (0.11) 3. 2.93 (0.10)   ANOVA F =1.24; P =0.30   1. 3.03 (0.14) 2. 2.97 (0.08) 3. 2.93 (0.10)   ANOVA F =3.91; P =0.03   1. 3.04 (0.10) 2. 2.97 (0.12) 3. 2.96 (0.09)   ANOVA F =2.86; P =0.07   1. 3.08 (0.12) 2. 2.96 (0.09) 3. 2.95 (0.08)   ANOVA F =8.79; P =0.001 | NA | Serum IgG was negatively associated with adequacy of social support in subjects with their peak level of job strain.  Furthermore, an almost significant negative correlation was found between job strain and availability of social support with serum IgG.  Job strain was positively associated with IgG, and lower social support was associated with higher IgG. | Adapted NOS (PA Modesti): 2-0-3 |
| Hui, Brain Behav Immun, 2007 | Sleep deprivation (SD); SD was defined as staying awake and engaging in mild social activity under guidance. The control group was required to sleep for at least 7 hours. | Mean (S) for:   1. Day 2 vs Day 1 SD 2. Day 2 vs Day 1 Control 3. Day 2 SD vs Control | 1. 287.89 (104.01) vs 236.22 (80.67) 2. 218.14 (111.20) vs 208.14 (108.49) 3. 287.89 (104.01) vs 218.14 (111.20)*   * P = 0.033 | 1. 1771 (291) vs 1453 (220) 2. 1387 (288) vs 1369 (205) 3. 1771 (291) vs 1387 (288)*   * P = 0.001 | 1. 194.78 (116.55) vs 157.98 (89.66) 2. 144.50 (65.85) vs 139.76 (50.23) 3. 194.78 (116.55) vs 144.50 (65.85)*   * P = 0.029 | IgA, IgG and IgM levels were higher in the SD group compared to the control group. Although levels remained within the reference range for IgA and IgM, they exceeded slightly for IgG in the SD group. | Cochrane risk of bias tool: some concerns |
| Oztürk, Sleep Res Online, 1999 | Sleep deprivation; SD group had to stay awake for 48 hours under supervision of two physicians, while the control subjects adhered to their usual sleep schedule. | Mean (SD) for:   1. SD vs control at baseline 2. SD vs control after 24h 3. SD vs control after 48h 4. SD vs control after 72h (recovery phase) | NA | 1. 837 (193) vs 1517 (156) 2. 802 (254) vs 1396 (160) 3. 880 (220) vs 1413 (102) 4. 869 (212) vs 1444 (121)   NS changes between 0-24h, 24-48h, 48-72h in either group | 1. 56.0 (20.0) vs 125 (63) 2. 49.5 (14.6) vs 116 (71) 3. 53.5 (15.3) vs 115 (71) 4. 61.8 (20.2) vs 117 (60)   NS changes between 0-24h, 24-48h, 48-72h in either group | Serum IgG and IgM levels of sleep deprived and control groups did not show any changes. | NIH assessment tool for before-after studies: 6/12 |
| Ruiz, Innate Immun, 2012 | Sleep deprivation (SD) (total and REM sleep deprivation); The non-SD group could sleep as usual, whereas REM-SD participants were woken at the first sign of REM sleep and kept awake for a certain time to avoid relapse into REM sleep. In the total SD group, participants had to stay awake continuously. | Description | Baseline IgA REM SD vs control group: 220 vs 160 (P = NS).  There was an effect of SD on IgA. IgA levels decreased by 6.6%, 6.9%, 4.9% and 4.6% each night during the four day period of REM SD when compared to baseline levels (P <0.01; P <0.01; P <0.03; P <0.04, respectively). Furthermore, IgA levels recovered after the 1^st^ night of recovery but dropped again after 2^nd^ and 3^rd^ nights of recovery (P <0.01) | No significant difference (data not shown) | No significant difference (data not shown) | In the REM SD group, IgA levels decreased during the entire period of SD in relation to baseline, although IgG and IgM levels were unchanged. | NIH assessment tool for before-after studies: 6/12 |
| Behr, J Clin Chem Clin Biochem, 1985 | Oral contraceptive (OCP) use | Mean (SD) for:   1. Women 19-39y without OCP 2. Women 19-48y with OCP 3. Women 40-60y without OCP | 1. 1767 (605) 2. 1518 (525) 3. 2131 (689) | 1. 11510 (2110) 2. 10560 (2060) 3. 10890 (1710) | 1. 1730 (579) 2. 1430 (563) 3. 1439 (600) | Women using OCPs had lower values of IgA, IgG and IgM compared to women not using OCPs.  However, no P-values were provided. | Adapted NOS (PA Modesti): 2-0-2 |
| Butler, J Clin Invest, 1973 | Methylprednisolone (16mg orally every 4h for either 3 or 5 days + antacid) compared to control group receiving equal amount of antacid only | Mean (net; percentage change) for:   1. Methylprednisolone vs Control posttreatment 2. Methyprednisolone posttreatment vs pretreatment 3. Control posttreatment vs pretreatment | 1. 1.38 vs 1.26 2. 1.38 vs 1.62 (-0.24; -16.3) 3. 1.26 vs 1.33 (-0.07; -5.8) | 1. 8.7 vs 12.2 2. 8.7 vs 11.2 (-2.5; -22.1) 3. 12.2 vs 12.4 (-0.2; -1.3) | 1. 1.39 vs 1.45 2. 1.39 vs 1.52 (-0.13; -6.5) 3. 1.45 vs 1.49 (-0.04; -2.0) | IgG levels were initially unchanged for a few days after methylprednisolone treatment, but decreased rapidly afterwards. A treatment duration of 5 instead of 3 days led to a slower recovery of IgG levels after study completion. | Cochrane risk of bias tool: high risk |
| Gómez, Am J Reprod Immunol, 1993 | Menstrual cycle | Mean (SD) for:   1. Women follicular vs luteal phase | 1. 358.19 (109.09) vs 368.59 (98.45) (P = NS) | NA | NA | No mentrual differences were observed for serum IgA. | Adapted NOS (PA Modesti): 1-0-3 |
| Goodwin, Int J Immunopharmacol, 1986 | Oral PGE1 analogue (misoprostol) 200µg 4 times daily for 2w vs control with placebo pills | Description | No significant changes in either group throughout the study (data not shown) | No significant changes in either group throughout the study (data not shown) | No significant changes in either group throughout the study (data not shown) | In either treatment group no differences in serum IgA, IgG, and IgM were observed. | Cochrane risk of bias tool: some concerns |
| Khorram, J Gerontol A Biol Sci Med Sci, 1997 | Dehydroepiandrosterone (DHEA) (50mg once daily for 20 weeks after 2 weeks of 50mg placebo) | Mean (SEM) after:   1. Placebo 2. 2 weeks of treatment 3. 10 weeks of treatment 4. 20 weeks of treatment | 1. 1869 (168) 2. 1934 (191) 3. 1860 (192) 4. 1914 (174) | 1. 11152 (757) 2. 11015 (515) 3. 10933 (675) 4. 11245 (860) | 1. 1216 (208) 2. 1187 (187) 3. 1223 (202) 4. 1259 (209) | Serum IgA, IgG and IgM levels were not affected over the course of the study. | NIH assessment tool for before-after studies: 4/12 |
| Klinger, Gynecol Endocrinol, 2000 | Oral contraceptive (OCP); Lovelle is desogestrel 0.15 mg plus ethinyl estradiol 0.02 mg; Valette is dienogest 2.0 mg plus ethinyl estradiol 0.03 mg | Mean (SD) for   1. Valette 10d vs baseline 2. Valette 21d vs baseline 3. Lovelle 10d vs baseline 4. Lovelle 21d vs baseline | 1. 2.04 (0.78) vs 2.20 (0.82)* 2. 2.09 (0.82) vs 2.20 (0.82) 3. 2.13 (0.58) vs 2.33 (0.61)* 4. 2.06 (0.55) vs 2.33 (0.61)*   * P <0.05 | 1. 11.91 (1.84) vs 12.53 (1.87)* 2. 12.20 (2.20) vs 12.53 (1.87) 3. 12.72 (2.97) vs 13.46 (3.07)* 4. 12.76 (2.85) vs 13.46 (3.07)*   * P <0.05 | 1. 1.51 (0.70) vs 1.69 (0.70)* 2. 1.60 (0.82) vs 1.69 (0.70) 3. 1.53 (0.56) vs 1.70 (0.71)* 4. 1.52 (0.56) vs 1.70 (0.71)*   * P <0.05 | Serum IgA, IgG, and IgM were reduced in both groups after 10 days. After 21 days these levels only remained reduced in the Lovelle group, and normalized in the Valette group. | NIH assessment tool for before-after studies: 7/12 |
| Otolorin, Acta Obstet Gynecol Scand, 1993 | Norplant (subdermal contraceptive) | Mean (SD) at:   1. Admission 2. One month 3. Three months 4. 12 months | 1. 216 (84) 2. 188 (95) 3. 194 (91) 4. 234 (78) | 1. 1368 (335) 2. 1494 (262)* 3. 1517 (331)* 4. 1462 (478)*   * P <0.05 | 1. 170 (98) 2. 187 (76) 3. 175 (74) 4. 180 (94) | IgG levels rise after one and three months of Norplant insertion, after 12 months the increase slows down but remains significant. | NIH assessment tool for before-after studies:  6/12 |
| Van Schoor, Clin Exp Allergy, 1997 | Corticosteroids | Median % change (range); P-value after 2 weeks treatment vs baseline for:   1. High dose oral prednisolone (40mg/day) 2. Low-dose oral prednisolone (10mg/day) 3. High-dose inhaled budesonide (3.2mg/day) | NA | 1. -13.5 (-54.4-+18.6); 0.066 2. -15.2 (-27.4--2.0); 0.043 3. No significant changes (data not shown) | NA | Treatment with low-dose oral prednisolone for 2 weeks lowered serum IgG levels. | Cochrane risk of bias tool: some concerns |
| Amagase, J Med Food, 2009 | L. Barbarum fruit juice (GoChi) (60 mL twice daily for 30 days) | Mean (SEM) for:   - 1. GoChi vs Placebo post-intervention   2. GoChi Post- vs Pre-intervention   3. Placebo Post- vs Pre-intervention | 1. 3,280.87 (275.60) vs 2,733.90 (191.66) 2. 3,280.87 (275.60) vs 2,709.23 (188.50) 3. 2,733.90 (191.66) vs 2,586.33 (230.38) | 1. 16.78 (0.50) vs 14.98 (0.63)* 2. 16.78 (0.50) vs 14.12 (0.52)* 3. 14.98 (0.63) vs 14.70 (0.58)   * P <0.05 | NA | GoChi treatment increased serum IgG levels compared to the placebo group and compared to the baseline level in the GoChi group. | Cochrane risk of bias tool: some concerns |
| Anderson, Am J Clin Nutr, 1980 | Ascorbate (1g daily for 1^st^ week, 2g daily for 2^nd^ week, 3g daily for 3^rd^ week) | Mean (SE) at/after:   1. Baseline 2. After 1g ascorbate (1st week) 3. After 2g ascorbate (2nd week) 4. After 3g ascorbate (3rd week) 5. 1 week after ascorbate cessation | 1. 262 (18) 2. 248 (19) 3. 240 (19) 4. 244 (18) 5. 242 (19) | 1. 1,788 (72) 2. 1,664 (93) 3. 1,620 (85) 4. 1,688 (73) 5. 1,696 (81) | 1. 266 (48) 2. 264 (47) 3. 242 (48) 4. 250 (46) 5. 254 (47) | Ascorbate did not influence serum immunoglobulin levels (no P-values provided). | NIH assessment tool for  before-after studies: 4/12 |
| Dlouhý, Ann Nutr Metab, 2008 | Consumption of trans fatty acids (TFA); habitual diet is the subject’s own diet at home (unknown composition), standard diet was a supervised diet for 1w followed by a 96-h TFA enriched diet | Mean (SD) for:   1. Point 1: habitual diet 2. Point 2: standard diet 3. Point 3: TFA-enriched diet 4. Point 4: habitual diet | 1. 1.926 (0.9781) 2. 1.967 (1.0047) 3. 1.841 (0.9383) 4. 1.920 (0.9754) | 1. 11.876 (2.0171) 2. 12.074 (2.2065) 3. 11.676 (2.1076) 4. 11.615 (2.0786) | 1. 1.191 (0.7098) 2. 1.244 (0.7982) 3. 1.156 (0.6944) 4. 1.175 (0.6891) | Throughout the study, there were no differences in serum IgA, IgG, and IgM levels. | NIH assessment tool for before-after studies: 6/12 |
| Farges, Br J Nutr, 2012 | Carotenoids (3 week depletion by eating less fruits and vegetables followed by 5 week repletion period with 3 carotenoid capsules a day and normal diet) | Mean (SEM) for:   1. Baseline 2. After carotenoid depletion 3. After carotenoid repletion | 1. 2.56 (0.1) 2. 2.58 (0.1) 3. 2.61 (0.1)   Two-way ANOVA: NS | 1. 11.1 (0.2) 2. 11.3 (0.2) 3. 11.1 (0.2)   Two-way ANOVA: NS | 1. 1.22 (0.06) 2. 1.23 (0.06) 3. 1.23 (0.06)   Two-way ANOVA: NS | Serum immunoglobulin levels were unaffected by the dietary intervention with carotenoids. | NIH assessment tool for before-after studies: 5/12 |
| Hartoma, Nutr Metab, 1979 | Serum zinc (µg/100 ml); subjects with low serum zinc received 220mg of zinc three times daily during 2 months | Mean (SD) for:   1. Low vs High serum zinc 2. Low serum zinc before vs after treatment | 1. 2.61 (0.59) vs 1.81 (0.64); P <0.10 2. 2.61 (0.59) vs 2.79 (0.68) | No significant difference (data not shown) | No significant difference (data not shown) | Trend of higher IgA levels in subjects with low serum zinc, although not significantly. IgG and IgM unaffected. | NIH assessment tool for before-after studies: 4/12 |
| Jayachandran, J Anti-Aging Med, 2000 | Ascorbate (200mg daily for 30, 60, or 90 days);  Participants divided in young (20-30y) and older (>60y) groups. | Mean (SD) for:   1. Ascorbate 30 days vs Control in young 2. Ascorbate 30 days vs Control in aged 3. Ascorbate 60 days vs Control in young 4. Ascorbate 60 days vs Control in aged 5. Ascorbate 90 days vs Control in young 6. Ascorbate 90 days vs Control in aged | 1. 2.57 (0.62) vs 2.55 (0.55) 2. 2.66 (0.64) vs 2.64 (0.51) 3. 2.59 (0.64) vs 2.55 (0.55) 4. 2.59 (0.84) vs 2.64 (0.51) 5. 2.62 (0.79) vs 2.55 (0.55) 6. 2.65 (0.93) vs 2.64 (0.51) | 1. 12.3 (1.34) vs 11.9 (1.73) 2. 8.29 (1.39) vs 7.54 (1.30) 3. 12.5 (1.46) vs 11.9 (1.73) 4. 9.19 (1.54) vs 7.54 (1.30) 5. 12.6 (1.38) vs 11.9 (1.73) 6. 10.8 (1.58) vs 7.54 (1.30) | 1. 1.65 (0.45) vs 1.62 (0.55) 2. 0.74 (0.26) vs 0.63 (0.22) 3. 1.72 (0.49) vs 1.62 (0.55) 4. 0.91 (0.33) vs 0.63 (0.22) 5. 1.82 (0.55) vs 1.62 (0.55) 6. 1.31 (0.50) vs 0.63 (0.22) | No association between ascorbate and serum immunoglobulin values was found for either age group. | Cochrane risk of bias tool: high risk |
| Kelley, Lipids, 1998 | Docosahexaenoic acid (DHA) (15g of DHASCO oil containing 40% DHA) vs control group with safflower oil in diet | Mean (SEM) for:   1. DHA vs Control after 30 days 2. DHA vs Control after 113 days | NR | 1. 86.0 (8.4) vs 85.1 (3.7) 2. 91.6 (8.4) vs 93.0 (8.2) | NR | No differences in IgG levels were observed by DHA supplementation. | Cochrane risk of bias tool: high risk |
| Kianbakht, Phytother Res, 2011 | Saffron (tablet of 100mg daily for 6 weeks) | Mean (SEM) for:   1. Saffron vs Placebo at baseline 2. Saffron vs Placebo after 3 weeks 3. Saffron vs Placebo after 6 weeks | 1. 344.4 (30.7) vs 355.9 (14) 2. 313.1 (27) vs 291.1 (16.4) 3. 323.8 (29.8) vs 274.1 (22.1) | 1. 1662.4 (69.1) vs 1608.7 (128.2) 2. 2065.5 (79.3)** vs 1578.1 (123.3) 3. 1558.6 (71) vs 1656.3 (72)   ** P<0.01 compared to baseline and placebo | 1. 184.3 (19.1) vs 177.2 (17.9) 2. 105.9 (9.1)** vs 173.1 (14.2) 3. 179.7 (22.7) vs 170.3 (26.6)   ** P<0.01 compared to baseline placebo | After 3 weeks, serum IgG levels were increased, whereas IgM was decreased in the saffron group, both compared to the baseline values and the placebo group (P<0.01). | Cochrane risk of bias tool: high risk |
| Kim, Nutrition, 2002 | Nutrient intake | Correlation coefficient for:   1. Dietary energy (kcal) 2. Protein 3. Fat 4. Carbohydrates 5. Fiber 6. Calcium 7. Phosphate 8. Iron 9. Vitamin A 10. Vitamin B1 11. Vitamin B2 12. Niacin 13. Vitamin C | 1. 0.7010* 2. -0.5827 3. -0.5075 4. 0.6652* 5. -0.1599 6. -0.4758 7. -0.1227 8. -0.5219 9. 0.3765 10. -0.2670 11. -0.3273 12. -0.2925 13. -0.5416   * P <0.05 | 1. -0.2190 2. -0.0168 3. -0.5009 4. -0.1434 5. 0.1300 6. -0.0663 7. 0.1265 8. 0.1611 9. 0.0725 10. -0.1360 11. -0.0611 12. 0.1114 13. -0.4301 | 1. 0.0718 2. -0.1867 3. -0.2959 4. 0.2839 5. -0.4152 6. -0.3155 7. -0.4330 8. -0.4063 9. 0.0228 10. -0.2750 11. -0.3134 12. -0.2444 13. -0.2494 | Serum IgA was positively correlated with dietary energy intake and consumption of carbohydrates. | Adapted NOS (PA Modesti): 2-0-3 |
| Kim, J Med Food, 2006 | Bacillus Polyfermenticus (Bispan) tablets vs placebo (yeast) tablets; participants used 2 tablets a day for 8 weeks | Mean (SD) in mg/dl for:   1. Bispan vs Placebo end of study 2. Bispan baseline vs end of study 3. Placebo baseline vs end of study | 1. 208.54 (31.09) vs 192.17 (31.22) 2. 197.92 (26.37) vs 208.54 (31.09) 3. 188.83 (27.34) vs 192.17 (31.22) | 1. 1308.76 (38.97) vs 1168.67 (68.95)* 2. 1208.74 (45.38) vs 1308.76 (38.97)* 3. 1192.58 (73.28) vs 1168.67 (68.95)   * P <0.05 | 1. 122.54 (37.11) vs 96.26 (17.95) 2. 115.58 (38.48) vs 122.54 (37.11) 3. 107.67 (21.47) vs 96.26 (17.95) | Bispan increases serum IgG levels after 8 weeks compared to the placebo group and baseline Bispan group values. | Cochrane risk of bias tool: some concerns |
| Lomax, Br J Nutr, 2012 | Synergy1 (a 50:50 mixture of long-chain inulin and shorter-chain oligofructose; considered to be prebiotics) vs maltodextrin (control); subjects consumed 2 sachets a day for 8 weeks | Median (25th-75th percentiles) for:   1. Placebo week 4 vs week 0 2. Prebiotic week 4 vs week 0 3. Prebiotic vs Placebo week 0 4. Prebiotic vs Placebo week 4 | 1. 2.07 (1.10-3.89) vs 2.80 (1.64-4.07) 2. 2.29 (1.53-3.92) vs 3.74 (2.82-4.34)* 3. 3.74 (2.82-4.34) vs 2.80 (1.64-4.07) 4. 2.29 (1.53-3.92) vs 2.07 (1.10-3.89)   * P <0.05 | 1. 27.38 (14.69-45.40) vs 16.34 (12.04-30.19)* 2. 28.17 (12.91-38.42) vs 20.43 (10.37-30.98) 3. 20.43 (10.37-30.98) vs 16.34 (12.04-30.19) 4. 28.17 (12.91-38.42) vs 27.38 (14.69-45.40)   * P <0.05 | 1. 0.90 (0.40-1.07) vs 0.96 (0.73-1.44)* 2. 0.88 (0.43-1.01) vs 0.98 (0.79-1.38)* 3. 0.98 (0.79-1.38) vs 0.96 (0.73-1.44) 4. 0.88 (0.43-1.01) vs 0.90 (0.40-1.07)   * P <0.05 | Throughout the study there were no differences in serum immunoglobulin levels between the Synergy1 and maltodextrin group. Within the Syngery1 group, IgA and IgM were lower in week 4 compared to week 0. Similar results were seen in the maltodextrin group for IgM, whereas IgG was higher in week 4 compared to week 0. | Cochrane risk of bias tool: some concerns |
| Marteau, Gastroenterol Clin Biol, 1997 | Lactobacillus strain La1; participants were randomized to consume twice daily 150g of milk which either did or did not contain La1 for 28 days | Mean (SD) for:   1. La1 after 28d vs baseline 2. Controls after 28d vs baseline 3. La1 vs control baseline 4. La1 vs control after 28d | 1. 1.85 (0.64) vs 1.76 (0.67)* 2. 2.46 (0.72) vs 2.35 (0.71) 3. 1.76 (0.67) vs 2.35 (0.71) 4. 1.85 (0.64) vs 2.46 (0.72)   * P = 0.02 | 1. 10.38 (0.88) vs 9.98 (0.86)  2. 12.58 (2.25) vs 12.62 (2.21)  3. 9.98 (0.86) vs 12.62 (2.21)  4. 10.38 (0.88) vs 12.58 (2.25) | 1. 1.12 (0.10) vs 1.08 (0.09) 2. 1.36 (0.24) vs 1.37 (0.24) 3. 1.08 (0.09) vs 1.37 (0.24) 4. 1.12 (0.10) vs 1.36 (0.24) | A small increase in serum IgA was observed in the La1 group after 28 days compared to baseline. No other differences were observed in either group. | Cochrane risk of bias tool: some concerns |
| O'Brien, Nutr Res, 1988 | 4 dietary periods based on egg and ascorbate consumption; all participants followed each diet for 12 weeks with 5 weeks rest in between:   - Basal = no eggs or ascorbate - EG = 3 eggs, but no ascorbate - AA = 1,5g ascorbate, no eggs - EGAA = 3 eggs and 1,5g ascorbate | Description (immunologic parameters were measured at 0, 6, and 12 weeks of each dietary period) | No changes (data not shown) | No changes (data not shown) | No changes (data not shown) | Serum concentrations of IgA, IgG and IgM were not influenced by consumption of eggs and ascorbate. | NIH assessment tool for before-after studies: 4/12 |
| Park, J Nutr Sci Vitaminol (Tokyo), 2004 | Resistant starch; participants received a resistant corn starch or regular corn starch (control group) supplement for 21 days; 40g of the supplement had to be taken daily mixed into 250ml water | Mean (SE) for:   1. Resistant starch end of study vs baseline 2. Regular starch end of study vs baseline | NA | 1. 1,149.8 (57.3) vs 1,079.8 (61.4)* 2. 1,209.6 (48.8) vs 1,222.2 (40.8)   * P <0.05 | NA | Resistant starch supplementation increased IgG levels after 21 days compared to baseline. No comparisons were made between the resistant starch and regular starch groups. | NIH assessment tool for before-after studies: 6/12 |
| Pongpaew, Arch Gerontol Geriatr, 1995 | Consumption of various nutrients | Correlation coefficient (P-value) for:   1. Calories 2. Protein 3. Carbohydrate 4. Fat 5. Fibre | 1. 0.1958 (NS) 2. 0.2543 (NS) 3. 0.1595 (NS) 4. 0.0523 (NS) 5. -0.2150 (NS) | 1. -0.1572 (NS) 2. -0.0775 (NS) 3. -0.1163 (NS) 4. -0.3632 (NS) 5. 0.1209 (NS) | 1. -0.2047 (NS) 2. 0.1135 (NS) 3. -0.0328 (NS) 4. -0.4034 (NS) 5. 0.1400 (NS) | There were no associations between macronutrients and serum immunoglobulin levels. | Adapted NOS (PA Modesti): 2-0-3 |
| Predy, J Clin Biochem Nutr, 2006 | COLD-fX (capsule formulated from the roots of North American ginseng) 2 capsules of 200mg each per day for 4 months compared to equal amount of rice powder (placebo) tablets | Mean (SD) for:   1. COLD-fX vs Placebo at baseline 2. COLD-fX vs Placebo after study completion 3. COLD-fX Baseline vs End of study 4. Placebo Baseline vs End of study | 1. 2 (0.7) vs 2.1 (1.0); P-value =0.80 2. 1.8 (0.6) vs 1.9 (0.8); P-value =0.77 3. 2 (0.7) vs 1.8 (0.6); P-value =0.03 4. 2.1 (1.0) vs 1.9 (0.8); P-value =0.20 | NA | NA | Serum IgA levels were significantly lower after study completion in de COLD-fX group compared to the baseline value. No difference between COLD-fX and placebo. | Cochrane risk of bias tool: some concerns |
| Prinz, Int J Vitam Nutr Res, 1977 | Ascorbic acid (in the experimental group participants were required to ingest 1g vitamin C per day during 11 weeks) | Mean (SD) for:   1. Control group end of study vs baseline 2. Experimental group end of study vs baseline | 1. 100.2 (41.9) vs 114.3 (39.2); P <0.005 2. 128.7 (59.4) vs 126.0 (57.9); P = NS | 1. 122.1 (28.4) vs 120.4 (35.8); P = NS 2. 109.4 (31.8) vs 93.9 (26.9); P = NS | 1. 173.2 (50.1) vs 184.1 (40.9); P = NS 2. 199.3 (64.9) vs 175.8 (62.0); P <0.005 | Serum IgA was decreased after 11 weeks in the control group. In the ascorbate group, IgM was increased at end of study compared to baseline. | NIH assessment tool for before-after studies: 6/12 |
| Sakem, BMC Med, 2013 | 25-hydroxyvitamin D levels (25(OH)D) (ng/ml), seasonal variation | Mean (SD) for:   1. 25(OH)D <10 2. 25(OH)D 10-20 3. 25(OH)D 21-29 4. 25(OH)D ≥30 | 1. 2.32 (0.10) 2. 2.12 (0.05) 3. 2.22 (0.05) 4. 2.12 (0.05)   P for trend = 0.12  P-value severely deficient (<10) and normal (≥30) = 0.036 | 1. 10.63 (0.25) 2. 10.05 (0.11) 3. 10.28 (0.10) 4. 10.28 (0.12)   P for trend = 0.24  P-value severely deficient (<10) and normal (≥30) = >0.05  Seasonal variation was established for IgG (P =0.001); IgG levels peak during August (together with 25(OH)D peak). | 1. 0.88 (0.05) 2. 0.94 (0.04) 3. 0.96 (0.03) 4. 0.98 (0.03)   P for trend = 0.09  P-value severely deficient (<10) and normal (≥30) = >0.05 | Serum IgA levels inversely correlate with 25(OH)D, whereas no association was found with IgG and IgM. IgG levels peak during August. | Adapted NOS (PA Modesti): 3-0-3 |
| Sierra, Anaerobe, 2010 | Breast-milk lactic acid bacteria L. Salivarius CECT5713;  Participants were randomized into a probiotic group (2 daily capsules of L. Salivarius CECT5713 with maltodextrins) and a control group (2 daily capsules of maltodextrins) for 4 weeks | Mean (SEM) for:   1. Probiotic week 2 vs week 0 2. Probiotic week 4 vs week 0 3. Control week 2 vs week 0 4. Control week 4 vs week 0 5. Probiotic vs control week 0 6. Probiotic vs control week 2 7. Probiotic vs control week 4 | 1. 923.6 (209.3) vs 1036.2 (213.2) 2. 1681.5 (471.0) vs 1036.2 (213.2)* 3. 936.4 (265.3) vs 1032.8 (310.8) 4. 907 (209.1) vs 1032.8 (310.8) 5. 1036.2 (213.2) vs 1032.8 (310.8) 6. 923.6 (209.3) vs 936.4 (265.3) 7. 1681.5 (471.0) vs 907 (209.1)   * P <0.05 | 1. 891.6 (210.3) vs 1050.1 (190.4) 2. 1829.9 (491.4) vs 1050.1 (190.4)* 3. 1015.4 (123.1) vs 1148.3 (139.4) 4. 1016.6 (196.9) vs 1148.3 (139.4) 5. 1050.1 (190.4) vs 1148.3 (139.4) 6. 891.6 (210.3) vs 1015.4 (123.1) 7. 1829.9 (491.4) vs 1016.6 (196.9)   * P <0.05 | 1. 305.1 (51.8) vs 351.5 (87.2) 2. 540.2 (165) vs 351.5 (87.2)* 3. 279.4 (21.8) vs 362.2 (80.5) 4. 261.1 (14.9) vs 362.2 (80.5) 5. 351.5 (87.2) vs 362.2 (80.5) 6. 305.1 (51.8) vs 279.4 (21.8) 7. 540.2 (165) vs 261.1 (14.9)   * P <0.05 | Compared to the beginning of treatment (week 0), after 4 weeks of treatment serum IgA, IgG and IgM were elevated in the probiotic group (P<0.05). No differences were observed between the probiotic and control groups. | Cochrane risk of bias tool: some concerns |
| Song, Eur J Clin Nutr, 2005 | Conjugated linoleic acid (CLA) (6 capsules of 500mg/day for 12 wk, followed by a 12wk wash out period); the control group received an equal amount of high oleic sunflower oil capsules | Graph with description | IgA levels were increased by 10% after 12 weeks of CLA supplementation; this increase continued after cessation and was still present in the washout period (P <0.05). In the reference group IgA decreased after 6 weeks (P <0.01) but then increased again after 12 weeks (P <0.05) | NA | IgM levels were increased in both the CLA and control group after supplementation, but the difference only reached significance in the CLA group (by 8 and 20%, at the 6-week and washout period, respectively, P <0.01). In the reference-oil group an increase was found after 12 weeks of (10%, P <0.05) | In both groups, IgA was increased after 12 weeks of supplementation and in the CLA group this increase remained significant after wash out. In the reference group IgA decreased after 6 weeks, but then increased again after 12 weeks. IgM levels were increased at all time points after CLA supplementation, but only after 12 weeks for the reference group. | NIH assessment tool for  before-after studies: 8/12 |
| Zhang, Synth Syst Biotechnol, 2018 | Probiotics; participants were randomized to drink a daily dose of 150ml probiotic (L. casei, L. paracasei, L. fermentum) or placebo yogurt drink for 12 weeks | Mean (SD) for:   1. Probiotic vs Placebo baseline 2. Probiotic vs Placebo after intervention   Difference mean (SD) for:   1. Probiotic vs Placebo (difference between after intervention-baseline) | 1. 2.15 (0.53) vs 2.12 (0.51); P =0.706 2. 2.23 (0.61) vs 2.10 (0.52); P =0.204 3. 0.08 (0.49) vs -0.01 (0.31); P =0.212 | 1. 11.84 (1.97) vs 12.08 (2.05); P =0.501 2. 12.10 (2.00) vs 11.97 (1.73); P =0.695 3. 0.26 (1.57) vs -0.10 (1.40); P =0.164 | 1. 1.07 (0.37) vs 1.10 (0.45); P =0.688 2. 1.14 (0.44) vs 1.12 (0.43); P =0.834 3. 0.07 (0.30) vs 0.02 (0.29); P =0.372 | IgA, IgG and IgM did not differ between the two groups, baseline and after the probiotics intervention. | Cochrane risk of bias tool: some concerns |
| Bahijri, Ther Adv Endocrinol Metab, 2015 | Ramadan fasting, values were compared between Ramadan and the preceding month (Shaban) and in both months at different time points during the day | Mean (SEM) for:   1. AM vs PM Shaban 2. AM vs PM Ramadan 3. AM Shaban vs Ramadan 4. PM Shaban vs Ramadan | 1. 1.86 (0.14) vs 1.91 (0.14); P =0.025 2. 1.85 (0.16) vs 2.10 (0.15); P <0.001 3. 1.86 (0.14) vs 1.85 (0.16); P =0.607 4. 1.91 (0.14) vs 2.10 (0.15); P =0.660 | 1. 12.37 (0.44) vs 12.67 (0.41); P =0.040 2. 11.33 (0.34) vs 12.56 (0.42); P <0.001 3. 12.37 (0.44) vs 11.33 (0.34); P =0.003 4. 12.67 (0.41) vs 12.56 (0.42); P =0.021 | 1. 0.873 (0.099) vs 0.893 (0.097); P =0.121 2. 0.873 (0.124) vs 0.945 (0.122); P =0.926 3. 0.873 (0.099) vs 0.873 (0.124); P =0.212 4. 0.893 (0.097) vs 0.945 (0.122); P =0.839 | During both Shaban and Ramadan, serum IgA and IgG levels were lower in the morning compared to the evening. Compared to Shaban, serum IgG levels were lower in Ramadan. | NIH assessment tool for before-after studies: 6/12 |
| Develioglu, J Int Med Res, 2013 | Ramadan fasting | Mean (SD) for:   1. One week before vs during the final week of Ramadan | NA | 1. 1291.5 (276.0) vs 1178.6 (230.3)*   * P-value =0.001 | 1. 117.8 (79.2) vs 116.1 (79.8) | IgG dropped during Ramadan (P=0.001), but remained within the reference range. | NIH assessment tool for  before-after studies: 5/12 |
| De Bruijn, Clin Sci (Lond), 1983 | Blood pressure (hypertension defined as mean arterial pressure (MAP) of ≥125 mmHg; normotension defined as MAP ≤90 mmHg) by age/sex | Mean (SEM)/P-value for:   1. Hypertensive vs Normotensive Male 2. Hypertensive vs Normotensive Female 3. Hypertensive vs Normotensive Male 40-54y 4. Hypertensive vs Normotensive Male 55-69y 5. Hypertensive vs Normotensive Female 40-54y 6. Hypertensive vs Normotensive Female 55-69y 7. Hypertensive vs Normotensive total 40-54y 8. Hypertensive vs Normotensive total 55-69y 9. Hypertensive vs Normotensive overall total | NA | 1. 11.1 (0.5) vs 11.1 (0.5)/ 0.98 2. 12.0 (0.6) vs 11.2 (0.5)/ 0.25 3. 10.9 (0.7) vs 10.2 (0.6)/ 0.44 4. 11.2 (0.7) vs 12.0 (0.8)/ 0.47 5. 11.6 (0.7) vs 11.5 (0.9)/ 0.93 6. 12.4 (0.9) vs 10.8 (0.6)/ 0.13 7. 11.3 (0.5) vs 10.9 (0.5)/ 0.58 8. 11.9 (0.6) vs 11.4 (0.5)/ 0.50 9. 11.6 (0.4) vs 11.1 (0.4)/ 0.38 | 1. 0.59 (0.06) vs 0.56 (0.06)/ 0.74 2. 0.73 (0.07) vs 0.66 (0.06)/ 0.50 3. 0.66 (0.09) vs 0.52 (0.06)/ 0.24 4. 0.51 (0.07) vs 0.59 (0.10)/ 0.49 5. 0.79 (0.11) vs 0.67 (0.08)/ 0.38 6. 0.67 (0.10) vs 0.65 (0.10)/ 0.90 7. 0.72 (0.07) vs 0.60 (0.05)/ 0.16 8. 0.60 (0.06) vs 0.63 (0.07)/ 0.75 9. 0.66 (0.05) vs 0.61 (0.04)/ 0.46 | No differences in mean IgG and IgM were found between the hypertensive and normotensive subjects. | Adapted NOS (PA Modesti): 2-0-2 |
| Gonzalez-Quintela, Clin Exp Immunol, 2008 | Blood pressure (high blood pressure was defined as ≥130/85 mm Hg or as use of antihypertensive medication) | Median (2.5^th^-97.5^th^ percentiles) for:   1. High blood pressure yes vs no | 1. 264 (89-583) vs 214 (84-427)*** | 1. 1110 (697-1841) vs 1040 (698-1502)** | 1. 129 (45-385) vs 141 (43-396) | High blood pressure was associated with higher IgA (P <0.001) and IgG (P <0.01) levels. | Adapted NOS (PA Modesti):  2-2-3 |
| Wang, J Hum Hypertens, 2018 | Blood pressure (hypertension was defined as a systolic blood pressure (SBP) of ≥140 mm Hg, a diastolic blood pressure (DBP) of ≥90 mm Hg, or as use of antihypertensive medication) | Mean SBP (95% CI) for:   1. 1^st^ Ig quintile 2. 2^nd^ Ig quintile 3. 3^rd^ Ig quintile 4. 4^th^ Ig quintile 5. 5^th^ Ig quintile   Mean DBP (95% CI) for:   1. 1^st^ Ig quintile 2. 2^nd^ Ig quintile 3. 3^rd^ Ig quintile 4. 4^th^ Ig quintile 5. 5^th^ Ig quintile   Ig, immunoglobulin | 1. 125.4 (119.2-132.0) 2. 125.3 (119.1-131.9) 3. 125.5 (119.3-132.1) 4. 126.1 (119.8-132.7) 5. 126.6 (120.3-133.3)   P ANCOVA: 0.02   1. 80.5 (76.2-85.0) 2. 80.4 (76.2-84.9) 3. 80.7 (76.4-85.2) 4. 81.4 (77.1-86.0) 5. 81.5 (77.1-86.0)   P ANCOVA: <0.001 | 1. 125.5 (119.2-132.0) 2. 124.8 (118.6-131.4) 3. 125.7 (119.4-132.3) 4. 125.9 (119.6-132.5) 5. 127.0 (120.7-133.7)   P ANCOVA: <0.0001   1. 80.9 (76.6-85.4) 2. 80.3 (76.1-84.8) 3. 80.7 (76.4-85.2) 4. 80.9 (76.6-85.4) 5. 81.6 (77.3-86.2)   P ANCOVA: <0.001 | 1. 126.3 (120.0-132.9) 2. 126.5 (120.2-133.1) 3. 125.5 (119.2-132.1) 4. 125.2 (119.0-131.8) 5. 125.1 (118.9-131.7)   P ANCOVA: <0.01   1. 81.0 (76.7-85.6) 2. 81.2 (76.9-85.8) 3. 80.6 (76.3-85.1) 4. 80.6 (76.3-85.1) 5. 80.7 (76.4-85.2)   P ANCOVA: 0.14 | SBP was highest in the higher IgA and IgG quintiles, while it was negatively associated with IgM. SBP was lower in the first 3 IgG quintiles compared to the 5^th^ quintile. DBP was highest in the 4^th^ and 5^th^ IgA and IgG quintiles. Additionally participants with hypertension had higher IgA and IgG, but lower IgM levels compared to normotensive participants. | Adapted NOS (PA Modesti):  3-2-3 |
| Gonzalez-Quintela, Clin Exp Immunol, 2008 | Body mass index (BMI), abdominal obesity (waist circumference >102cm in males, >88cm in females) | Median (2.5^th^-97.5^th^ percentiles) for:   1. Overweight vs normal weight 2. Obese vs normal weight 3. Abdominal obesity yes vs no | 1. 238 (90-588) vs 233 (80-531) 2. 270 (87-583) vs 233 (80-531)** 3. 270 (98-585) vs 232 (88-519)***   Adjusted for age, sex, alcohol intake, smoking status and metabolic syndrome, abdominal obesity was positively associated with IgA (P = 0.02) | 1. 1095 (734-1760) vs 1050 (713-1852) 2. 1130 (676-1777) vs 1050 (713-1852)* 3. 1130 (674-1789) vs 1085 (726-1755) | 1. 126 (46-370) vs 141 (42-412) 2. 129 (40-394) vs 141 (42-412) 3. 127 (39-397) vs 136 (46-389) | Obese individuals had higher IgA (P <0.01) and IgG (P <0.05) levels compared to people with a normal weight, abdominal obesity was also associated with higher IgA (P <0.001). | Adapted NOS (PA Modesti):  2-2-3 |
| Pongpaew, Arch Gerontol Geriatr, 1995 | Anthropometric measures | Correlation coefficient (P-value) for:   1. Weight 2. Height 3. BMI 4. Triceps skinfold thickness 5. Subscapular skinfold thickness 6. Arm circumference 7. Mid-arm muscle circumference | 1. 0.1575 (NS) 2. 0.2980 (NS) 3. 0.1071 (NS) 4. 0.0940 (NS) 5. -0.1339 (NS) 6. 0.0317 (NS) 7. -0.0209 (NS) | 1. -0.2118 (NS) 2. -0.1056 (NS) 3. -0.1862 (NS) 4. -0.0175 (NS) 5. -0.1219 (NS) 6. -0.2337 (NS) 7. -0.2101 (NS) | 1. 0.1295 (NS) 2. -0.1879 (NS) 3. 0.2488 (NS) 4. 0.3728 (P <0.05) 5. 0.2269 (NS) 6. 0.2661 (NS) 7. 0.1184 (NS) | A positive correlation was found between triceps skinfold thickness and serum IgM. | Adapted NOS (PA Modesti): 2-0-3 |
| Dufour, Int Immunopharmacol, 2005 | Amoxicillin/clavulanic acid (AMC); participants received 1g AMC orally twice a day for 5 days (starting on day 0) | Figure provided with mean (SEM) for days -13, -1, 5, 19, 61 | Mean (SEM) did not change throughout study | Mean (SEM) was significantly decreased at day 19 compared to day -13:  Day -13: 10.85 (1.84)  Day 19: 9.99 (1.51) | Mean (SEM) did not change throughout study | IgG significantly decreased between day -13 and 19, although the values remained within the reference range of the assay. | NIH assessment tool for before-after studies: 6/12 |
| Van Larebeke, Int J Environ Health Res, 2003 | Residence area in La Louvière (with respect to air pollution); adjusted for age, sex and smoking status | Mean (SD) for:   1. Peripheral area 2. Intermediate green 3. La Louviere Steelworks 4. Power Plant-Landfill | 1. 239 (106) 2. 245 (108) 3. 256 (128) 4. 218 (135)   ANCOVA P-value for difference NS  In the more exposed areas compared to the peripheral area, more people had IgA levels ≥300 mg/dl (P <0.02). | 1. 1,171 (341) 2. 1,207 (267) 3. 1,181 (238) 4. 1,123 (370)   ANCOVA P-value for difference NS | 1. 125 (93) 2. 128 (64) 3. 117 (52) 4. 145 (91)   ANCOVA P-value for difference NS | Although higher IgA levels were more prevalent in the more polluted areas of La Louvière, mean IgA, IgG, and IgM levels did not differ between the areas. | Adapted NOS (PA Modesti): 2-2-3 |
| Kim, Nutrition, 2002 | Iron-deficiency related biochemical indices | Correlation coefficient for:   1. Serum iron 2. Total iron binding capacity 3. Ferritin 4. Transferrin | 1. -0.1089 2. 0.2740 3. -0.0844 4. 0.3523 | 1. -0.2259 2. 0.1799 3. -0.0954 4. 0.4699 | 1. 0.1922 2. 0.5127 3. 0.2874 4. 0.6272*   * P <0.05 | Serum transferrin was positively correlated with serum IgM. | Adapted NOS (PA Modesti): 2-0-3 |
| Modica, Expl Clin Immunogenet, 1989 | HLA-DR phenotype | Mean (SD) for:   1. HLA-DR1 2. HLA-DR2 3. HLA-DR3 4. HLA-DR4 5. HLA-DR5 6. HLA-DRw6 7. HLA-DR7 8. HLA-DR3+ 9. HLA-DR3- 10. HLA-BB, DR3+ 11. HLA-B8, DR3- | 1. 2.4 (1.0) 2. 2.4 (1.1) 3. 2.0 (0.7) 4. 2.3 (0.7) 5. 2.3 (1.1) 6. 2.1 (0.8) 7. 2.3 (0.9) 8. 2.0 (0.7) 9. 2.3 (1.0) 10. 1.8 (0.6) (P <0.05 for difference between HLA-B8, DR3+ and - subjects) 11. 2.3 (0.9) | 1. 11.8 (2.9) 2. 13.0 (4.6) 3. 11.7 (2.6) 4. 11.4 (2.4) 5. 12.4 (3.2) 6. 11.4 (2.6) 7. 12.0 (3.4) 8. 11.7 (2.6) 9. 12.0 (3.3) 10. 11.8 (2.2) 11. 11.9 (3.3) | 1. 1.7 (1.4) 2. 1.9 (1.9) 3. 1.4 (0.6) 4. 2.1 (1.4) 5. 1.7 (1.3) 6. 1.6 (0.7) 7. 1.8 (1.6) 8. 1.4 (0.6) 9. 1.8 (1.4) 10. 1.5 (0.8) 11. 1.7 (1.3) | HLA-DR3+ individuals displayed the lowest serum IgA levels, but the difference between HLA-DR3+ and - individuals only reached significance when the IgA levels were also analyzed according to the presence or absence of HLA-B8. | Adapted NOS (PA Modesti): 3-0-3 |
| Röcker, Isr J Med Sci, 1978 | Intermittent heat exposure in a sauna bath; participants were exposed to sauna heat for 4 hours with a 15 minute interval every hour at room temperature; there was a 45 min pre-exposure (control period) and after the 4h exposure there was a 180 min post-heat-stress (recovery) period | Median (25th- 75th percentile) for:   - 1. Immediately before heat exposure   2. Difference 10 min after dehydration   3. Difference 90 min after dehydration   4. Difference 180 min after dehydration   Compared to time point 1  * P 0.01-0.05; ** P <0.01; *** P <0.001 | Women   1. 1.60 (1.23-1.86) 2. +0.13 (+0.05-+0.20)*** 3. +0.06 (0.00-+0.13)* 4. +0.09 (+0.05-+0.22)**   Men   1. 1.83 (1.71-2.32) 2. +0.25 (+0.17-+0.28)*** 3. +0.18 (+0.13-+0.22)*** 4. +0.14 (+0.08-+0.26)*** | Women   1. 9.58 (9.18-12.36) 2. +1.28 (+0.75-+1.63)*** 3. +1.12 (+0.52-+1.44)*** 4. +0.46 (+0.20-+1.10)***   Men   1. 11.01 (10.03-12.63) 2. +1.57 (+1.25-+1.71)*** 3. +1.08 (+0.59-+1.79)** 4. +1.09 (+0.69-+1.74)*** | Women   1. 2.09 (1.45-2.32) 2. +0.13 (+0.06-+0.20)*** 3. +0.12 (0.00-+0.25)* 4. +0.11 (0.00-+0.25)*   Men   1. 1.70 (1.37-1.83) 2. +0.11 (+0.08-+0.28)*** 3. +0.06 (+0.05-+0.24)*** 4. +0.11 (+0.06-+0.15)*** | There were no differences in serum immunoglobulins between men and women. IgA, IgG, and IgM were all increased during heat exposure compared to the pre-exposure control period. | NIH assessment tool for before-after studies: 4/12 |
| Roseman, Nature, 1975 | Intelligence (Weschler adult intelligence scale; WAIS score) | Correlation coefficient (P-value) for:   1. Total WAIS score   Correlation coefficient (P-value) for:   1. Average score of 4 WAIS subsets (information, vocabulary, digit symbol, picture arrangement) | 1. r = 0.161 (P =NS) 2. r = -0.121 (P =NS) | 1. r = -0.332 (P <0.005) (after adjustment for age, race and sex r = -0.296; P <0.001) 2. r = -0.109 (P <0.02) | 1. r = 0.085 (P =NS) 2. r = 0.027 (P =NS) | There was a negative correlation between total and subsets of WAIS score with serum IgG. | Adapted NOS (PA Modesti): 3-2-2 |
| Toshkov, Folia Haematol, 1974 | AB0 blood group | Description | There were no significant differences by blood group type (data not shown) | There were no significant differences by blood group type (data not shown) | There were no significant differences by blood group type (data not shown) | AB0 blood group does not influence serum immunoglobulins. | Adapted NOS (PA Modesti): 3-0-2 |
| Veys, Clinica Chimica Acta, 1973 | External temperature | Mean (2.5th-97.5th percentile) in:   1. Blood samples at the day of drawing 2. Samples after storage of sera for 3-4 weeks at -20 degrees 3. Samples after storage of sera for 3 months at -20 degrees | 1. 208 (79-631) 2. 241 (77-758) 3. 193 (72-518) | 1. 1,275 (796-2,044) 2. 1,250 (766-2,057) 3. 1,233 (776-1,958) | 1. 74 (30-200) 2. 71 (27-186) 3. 67 (24-190) | IgM levels were significantly lower in the serum stored for 3 months compared to the freshly drawn serum values. IgA was also significantly lower in the 3-month-old serum compared to the fresh samples and those stored for 3-4 weeks. | Adapted NOS (PA Modesti):  3-0-2 |
| IgA, immunoglobulin A; IgG, immunoglobulin G; IgM, immunoglobulin M; SD, standard deviation; SE(M), standard error (of the mean); NA, not applicable; NS, not significant; NR, not reported.  ^$^Results are shown in alphabetical order of first author per determinant. | | | | | | | |

Records identified through database searching (n = 18 660)

Additional records identified through other sources (n = 16)

Records after duplicates removed (n = 9758)

Records excluded based on title and abstract (n = 9532)

Records screened (n = 9758)

Full-text articles excluded (n = 109)

- No general population (n = 19)
- Systematic review (n = 3)
- Letter to editor (n = 2)
- Full text not found despite contacting first authors (n = 41)
- No abstract (n = 22)
- Foreign language (n = 8)
- Exclusively participants <18y in study population or no age provided (n = 8)
- No data on immunoglobulins provided (n = 2)
- No determinant described (n = 3)
- Duplicate (n = 1)

Full-text articles assessed for eligibility (n = 226)

Studies included in qualitative synthesis (n = 117)

Studies included in quantitative synthesis (meta-analysis) (n = 28)

**Supplementary Figure S1. Flowchart of 117 included studies on the determinants of serum immunoglobulin levels**


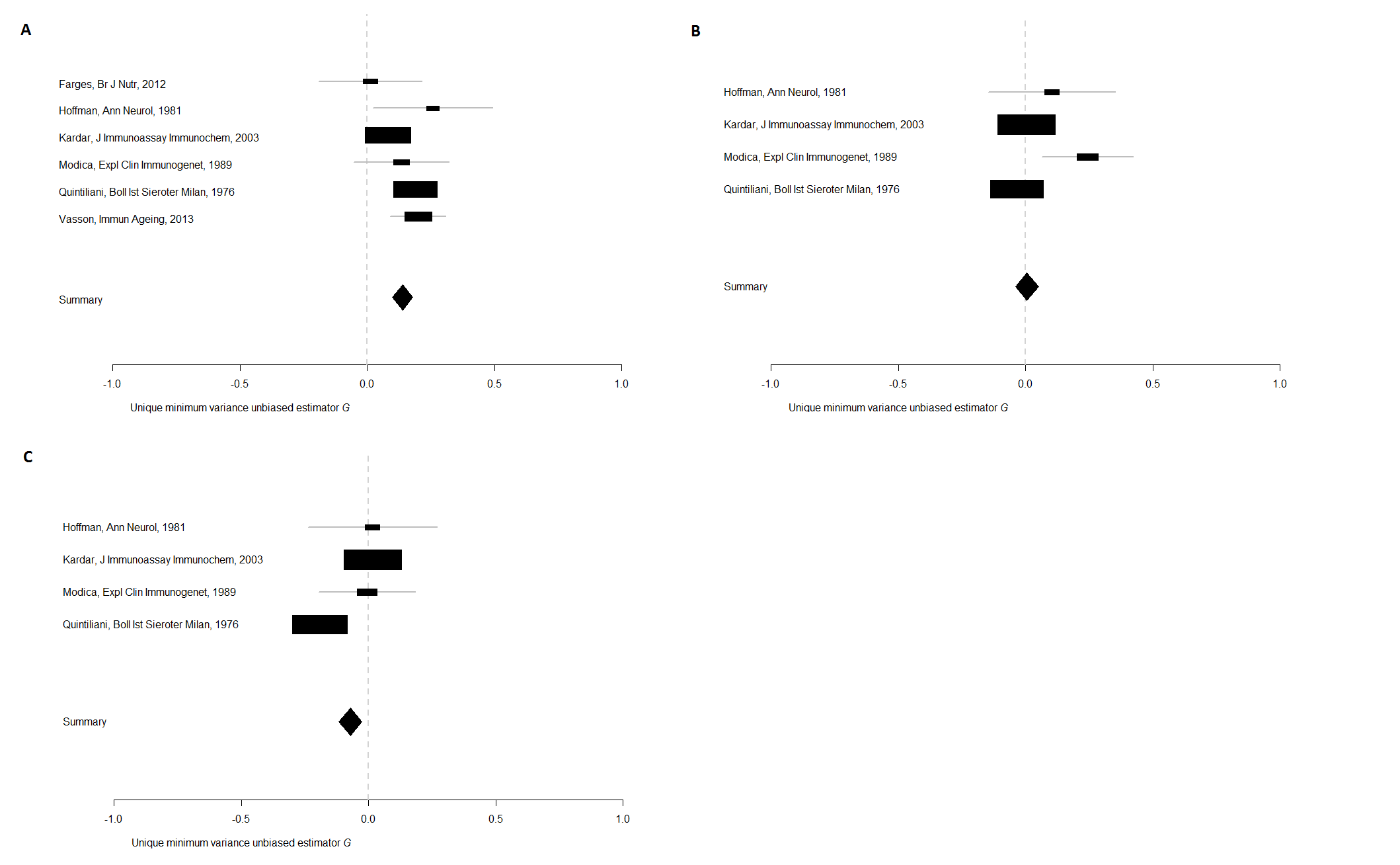


**Supplementary Figure S2. Forest plots for the correlation between age and serum immunoglobulin levels.**

1. Correlation of age with serum immunoglobulin A (IgA) (g/l)
2. Correlation of age with serum immunoglobulin G (IgG) (g/l)
3. Correlation of age with serum immunoglobulin M (IgM) (g/l)

The closed squares with horizontal lines depict the correlation coefficients with 95% confidence intervals for age and serum immunoglobulins. The diamonds depict the pooled correlation coefficient as estimated by the Olkin-Pratt fixed effect model.


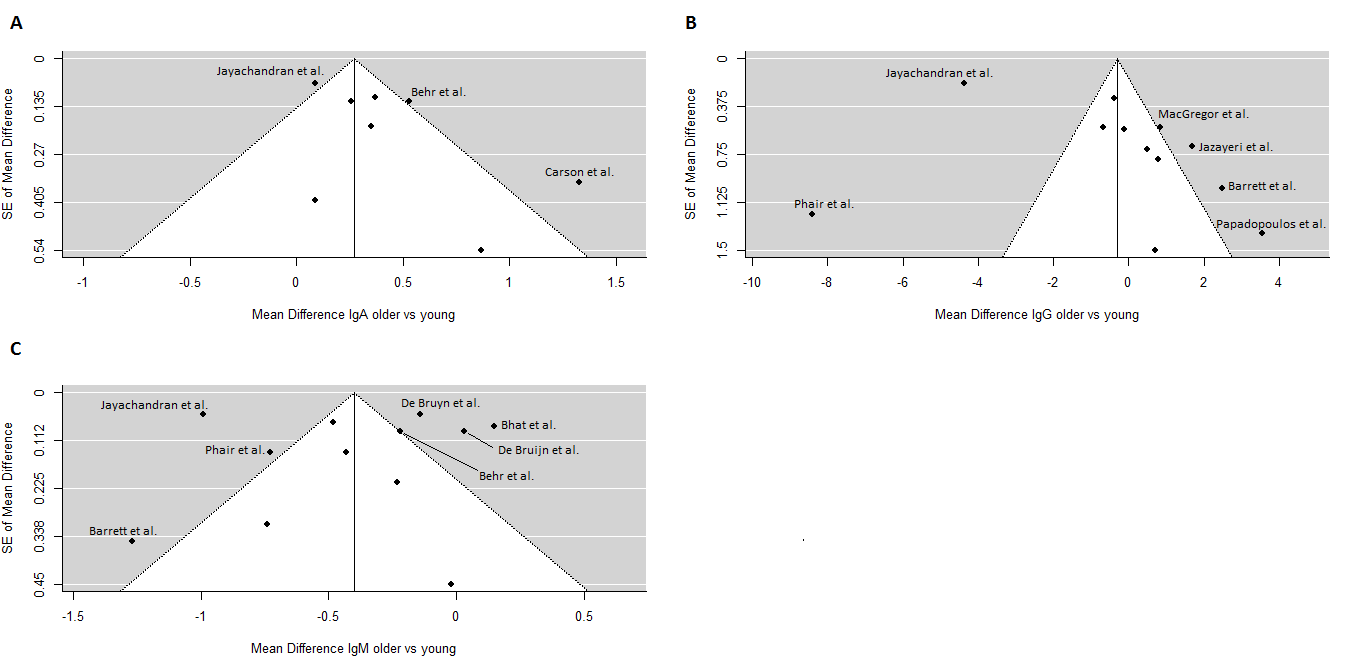


**Supplementary Figure S3. Funnel plots for the association between age and serum immunoglobulin levels.**

1. Funnel plot for the association of age with serum immunoglobulin A (IgA) (g/l); P-value for funnel plot asymmetry (Egger test) = 0.0024
2. Funnel plot for the association of age with serum immunoglobulin G (IgG) (g/l); P-value for funnel plot asymmetry (Egger test) = 0.6134
3. Funnel plot for the association of age with serum immunoglobulin M (IgM) (g/l); P-value for funnel plot asymmetry (Egger test) = 0.4280

The mean differences in serum immunoglobulin levels between older (≥45 years) and young (<45 years) subjects are plotted against the standard error (SE) of the mean differences. The vertical line depicts the summary mean difference with the triangle legs as its 95% confidence interval.


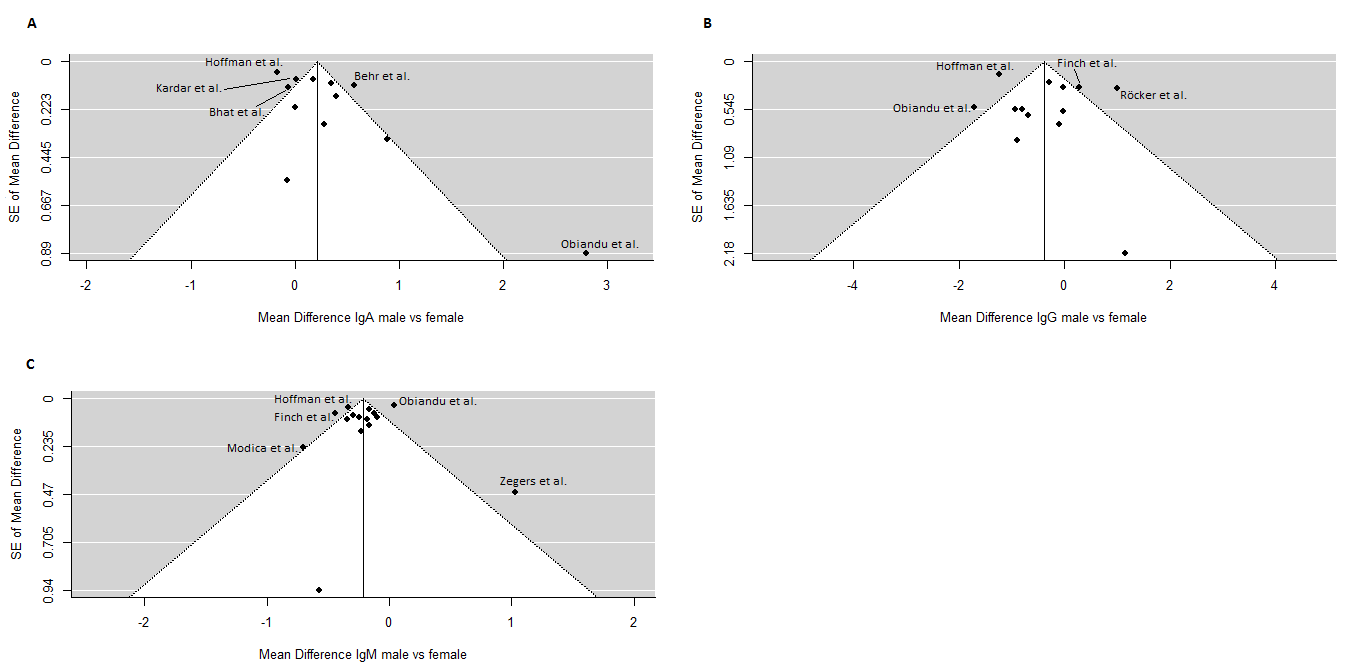


**Supplementary Figure S4. Funnel plots for the association between sex and serum immunoglobulin levels.**

1. Funnel plot for the association of sex with serum immunoglobulin A (IgA) (g/l); P-value for funnel plot asymmetry (Egger test) = 0.0128
2. Funnel plot for the association of sex with serum immunoglobulin G (IgG) (g/l); P-value for funnel plot asymmetry (Egger test) = 0.9891
3. Funnel plot for the association of sex with serum immunoglobulin M (IgM) (g/l); P-value for funnel plot asymmetry (Egger test) = 0.8532

The mean differences in serum immunoglobulin levels between men and women are plotted against the standard error (SE) of the mean differences. The vertical line depicts the summary mean difference with the triangle legs as its 95% confidence interval.


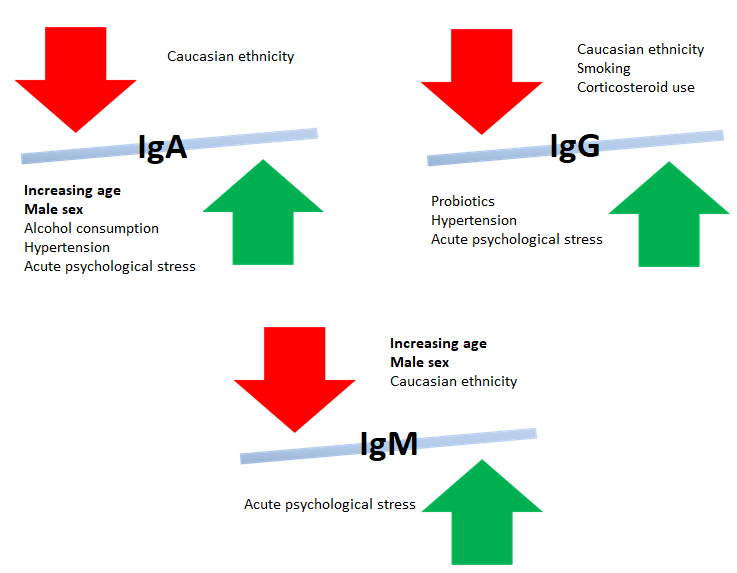


**Supplementary Figure S5. Summary of identified determinants.**

Red arrows depict factors associated with lower serum immunoglobulin levels, green arrows depict factors associated with higher serum immunoglobulin levels. In bold are the determinants that were included in the meta-analyses.
